# Ring-opening polymerization-induced crystallization-driven self-assembly of poly-L-lactide-*block*-polyethylene glycol block copolymers (ROPI-CDSA)

**DOI:** 10.1038/s41467-020-18460-2

**Published:** 2020-09-17

**Authors:** Paul J. Hurst, Alexander M. Rakowski, Joseph P. Patterson

**Affiliations:** Department of Chemistry, University of California, Irvine, Irvine, CA 92697-2025 USA

**Keywords:** Polymer synthesis, Self-assembly

## Abstract

The self-assembly of block copolymers into 1D, 2D and 3D nano- and microstructures is of great interest for a wide range of applications. A key challenge in this field is obtaining independent control over molecular structure and hierarchical structure in all dimensions using scalable one-pot chemistry. Here we report on the ring opening polymerization-induced crystallization-driven self-assembly (ROPI-CDSA) of poly-L-lactide-*block*-polyethylene glycol block copolymers into 1D, 2D and 3D nanostructures. A key feature of ROPI-CDSA is that the polymerization time is much shorter than the self-assembly relaxation time, resulting in a non-equilibrium self-assembly process. The self-assembly mechanism is analyzed by cryo-transmission electron microscopy, wide-angle x-ray scattering, Fourier transform infrared spectroscopy, and turbidity studies. The analysis revealed that the self-assembly mechanism is dependent on both the polymer molecular structure and concentration. Knowledge of the self-assembly mechanism enabled the kinetic trapping of multiple hierarchical structures from a single block copolymer.

## Abstract (web summary)

A key challenge in the self-assembly of block copolymers is obtaining independent control over molecular structure and hierarchical structure in all dimensions using scalable one-pot chemistry. Here the authors show the ring opening polymerization-induced crystallization-driven self-assembly of poly-L-lactide-*block*-polyethylene glycol block copolymers into 1D, 2D and 3D nanostructures.

## Introduction

Self-assembly of block copolymers (BCP) in solution has received significant interest in the areas of drug delivery, medical imaging, catalysis, and templated synthesis^1,2^. The properties and performance of these materials are intrinsically linked to both their molecular and hierarchical structure^3^. Consequently, research in this area has been focused on providing a fundamental understanding of how the molecular structure and assembly environment can be tailored to control the hierarchical assembly process^4^. Self-assembly processes can be either thermodynamically or kinetically controlled^5,6^. Thermodynamically controlled processes will adopt the hierarchical structure with the lowest free energy regardless of the starting conformation of the BCPs. Thermodynamically controlled processes tend to result in predictable and well-defined structures. However, their application is limited by their sensitivity to changes in the environment, which can significantly alter the formed structures^7,8^. Kinetically controlled processes tend to result in stable structures, but the final structures can be highly sensitive to the assembly method^5,7,9^. While the final structural dependence on assembly methodology offers the flexibility to form multiple hierarchical structures from a single BCP, it necessitates strict control of the synthetic conditions^3,7,10^.

Typically, BCP self-assembly is achieved via direct dissolution, solvent-switch, and thin-film hydration methods^11–13^. However, with these methods, it is challenging to control the formation of anisotropic structures, they involve multi-step processes that are difficult to scale up reproducibly, and result in low concentration BCP solutions (typically ≤1% solids w/w)^14–16^. To address these challenges, crystallization-driven self-assembly (CDSA) and polymerization-induced self-assembly (PISA) have emerged as promising alternatives. CDSA utilizes BCPs with a semi-crystalline core-forming block to form anisotropic 1D and 2D structures with high precision, where crystallization of the BCP core is the dominant driving force of self-assembly^17–23^. Whereas amorphous BCPs typically assemble into spheres, worms, and vesicles^14,24^, crystalline BCPs will typically result in morphologies with low curvature such as 2D platelet lamellae or 1D nanorods^15^, which introduces anisotropy to the system. In CDSA, the insoluble block is typically crystallized by dissolving the polymer in a selective solvent to facilitate /induce crystallization^18,24^, often utilizing heat-cool cycles to control the crystallization process^15,23,24^. However, as with traditional self-assembly methods, CDSA typically occurs in dilute solutions (~1% solids w/w)^14,20,24^. The PISA method utilizes controlled polymer chemistry to generate the BCP directly in the selective solvent system^11,13,14,25–27^. Conceptually, a homopolymer is chain-extended with a co-monomer and as the core-forming block grows it becomes increasingly insoluble, triggering self-assembly of the BCP. PISA is a scalable method that affords control over the BCP structure at high concentrations (10–50% solids w/w)^14,25,28–30^. To date, most PISA processes have been reversible-deactivation radical polymerizations (RDRP)^1,2,11,14,31^, primarily reversible addition-fragmentation chain-transfer polymerization (RAFT-PISA)^11,14,31–34^. However, PISA can theoretically be extended to all types of living polymerizations and has been demonstrated with living anionic polymerization^35^, ring-opening metathesis (ROMP) of norbornenes^13,29^, radical ring-opening copolymerization (rROP) of cyclic ketenes^36^, and ring-opening polymerization (ROP) of N-carboxyanhydrides^37^. PISA has also been combined with CDSA, termed polymerization-induced crystallization-driven self-assembly (PI-CDSA), to generate crystalline self-assemblies in high concentrations (10–25% solids w/w)^15,16,38^. Currently, PI-CDSA has been demonstrated for sequential living anionic polymerization^15,16^, and the ring-opening metathesis of organometallic polymers^38^. Polylactones, being semi-crystalline in nature, are excellent candidates for the development of a fully organic PI-CDSA to produce biocompatible, biodegradable BCP materials at high concentration in a scalable process. Polylactones, including poly(L-lactide) (PLLA) are the most widely known class of sustainable polymers^39^, and are incorporated into multiple FDA-approved formulas^40–42^. To date, ROP is the only controlled method to synthesize polylactone-based BCPs^2,28^. Thus, the development of a ring-opening PI-CDSA (ROPI-CDSA), would address the production limitations of polylactone-based crystalline-nanoparticles facilitating their use commercially.

The absence of ring-opening PI-CDSA of lactones is likely due to the stringent requirements for ROP reactions and the limited monomer/polymer/solvent combinations available^39,43,44^. Foremost, ROP of lactones cannot occur in protic solvents, as these solvents would compete with initiation species. However, some aprotic organic solvents, including aromatics like toluene, have induced crystallization in PLLA at room temperature^45^.

Here, we present the ring opening PI-CDSA of poly-(L-lactide)-*block*-polyethylene glycol (PLLA-*b*-PEG) BCPs in toluene. Kinetics studies of the polymerization and self-assembly show that ROPI-CDSA results in a non-equilibrium assembly process. Structural and morphological evolution of self-assembly is tracked with cryogenic-transmission electron microscopy (cryo-TEM), wide-angle X-ray scattering (WAXS), UV/Vis spectroscopy, and Fourier transform infrared (FTIR) spectroscopy, revealing a hierarchical (1D→2D→3D) growth mechanism. The data reveal that the assembly process and the final meta-stable structures can be controlled by alterations in both BCP molecular structure and concentration. Knowledge of the mechanism enables the trapping of materials with different structures and dimensionalities.

## Results

### ROPI-CDSA design and synthetic parameters

**Fig. 1:**
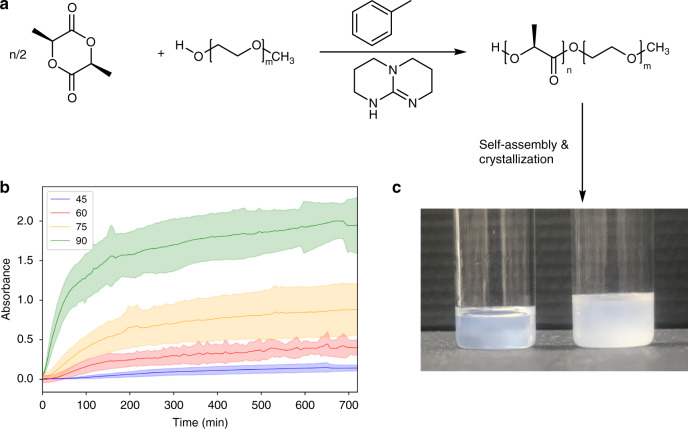
ROPI-CDSA scheme and self-assembly kinetics. **a** ROPI-CDSA scheme. **b** Turbidity measurements (UV/Vis 600 nm) for PLLA DP 45, 60, 75, and 90 at 10% solids w/w, with PEG DP 45 standard error calculated from 3 runs. The data show that the self-assembly kinetics vary as a function of PLLA DP, where increasing DP results in a faster rate of self-assembly. **c** Photographs showing the difference in turbidity for samples **9** (PLLA_45_-*b*-PEG_45_ 10% solids w/w left) and **15** (PLLA_90_-*b*-PEG_45_ 10% solids w/w right).

ROPI-CDSA experiments were performed using L-lactide as the monomer, mono-functional polyethylene glycol (mPEG_45_) as the initiator, toluene as the solvent and triazabicyclodecene (TBD) the catalyst (Fig. 1a)^44,46–48^. While other organocatalysts can achieve better dispersity (Ð) than TBD, they require high catalyst loading (1–10% mol vs 0.1% mol) and longer reaction times^48^. TBD is a highly active ROP catalyst that can achieve high conversion in seconds^39,47^. If reaction mixtures are left too long the Ɖ will broaden from transesterification^47^. Therefore, to limit transesterification reactions were quenched after 90 s for concentrations of 10% solids w/w and 60 s for concentrations greater than 10% solids w/w. As shown in Table 1, a library of 19 polymers was synthesized with a variation in the degree of polymerization (DP) of the PLLA block and total solids concentration (Supplementary Tables 1 and 2 for additional information on synthesis). For PLLA_10_-*b*-PEG_45_, **1**–**4** (5–20% solids w/w), no self-assembly was observed visually (i.e., solutions remain clear/no observed turbidity) or by cryo-TEM. At 20% solids w/w (PLLA_45_-*b-*PEG_45_ **10**, PLLA_90_-*b*-PEG_45_ **16**, and PLLA_135_-*b*-PEG_45_ **19**), control over Ð worsens, most likely due to transesterification from higher TBD solution concentrations (see also Supplementary Table 1). The 20% solids w/w samples macroscopically phase separate forming distinct solution and gel-like phases. Above 20% solids w/w, mPEG_45_ is not fully soluble in toluene. At 20% solids, limited PEG solubility may have also contributed to transesterification. For samples **5–19**, the polymerizations reached >92% (Supplementary Fig. 1) conversion within 60–90 s and resulted in well-defined (Ð < 1.2) BCP (Supplementary Fig. 2). The resulting solutions became turbid at various time points, ranging from during the polymerization for **16** (PLLA_90_-*b*-PEG_45_ 20% solids w/w) and **19** (PLLA_135_-*b*-PEG_45_) to more than 24 h post-polymerization for **5** (PLLA_25_-*b*-PEG_45_ 10% solids w/w) and **7** (PLLA_45_-*b*-PEG_45_ 5% solids w/w). This indicates that in most instances the relaxation time is significantly longer than the polymerization time and is dictated by the molecular structure of the BCP. Here, relaxation time refers to the time taken for the assembled structures to reach a low energy configuration and stop reorganizing. Control experiments were performed by synthesizing PLLA homopolymers in dichloromethane (where PLLA is fully soluble) and toluene (where PLLA is partially soluble), using ethanol as an initiator. In dichloromethane, >95% conversion was achieved in 90 s, whereas in toluene, only 25 and 28% conversion were achieved after 90 s for PLLA_45_ and PLLA_90_, respectively (Supplementary Table 3). This demonstrates that the PEG chains promote the polymerization by increasing the solubility of the growing PLLA chain in toluene. This is similar to previous PISA reports which have shown that self-assembly during polymerization can enhance the rate of polymerization of the selective block^14^. A second control was performed to determine if the polymerization is required for initiation of the self-assembly process, PLLA-*b*-PEG BCPs synthesized in dichloromethane were purified and re-dispersed directly in toluene. The polymers did not fully dissolve/disperse even when left over a period of months, indicating that direct polymerization in toluene is required to form stable assemblies at room temperature and that the structures formed in ROPI-CDSA are under kinetic control. Heat-cool cycles or a solvent-switch would likely also lead to the formation of stable assemblies, however, the purpose of the second control was to determine if the samples are pathway dependent. Pathway dependence indicates that it should be possible to control the ROPI-CDSA structures by modification of both the thermodynamics and kinetics of the process (see “Discussion” section on non-equilibrium PISA).

**Table 1:** Molecular characterization of PLLA-*b*-PEG BCPs synthesized via ROPI-CDSA.

Polymer	DP	% w/w	M_n_ (Da)^a^	Ð^b^
1	10	5.0	2700	1.07
2	10	7.5	2700	1.07
3	10	10.0	2700	1.08
4	10	20.0	2700	1.09
5	25	10.0	3700	1.11
6	25	20.0	3700	1.15
7	45	5.0	5200	1.12
8	45	7.5	5400	1.12
9	45	10.0	5300	1.16
10	45	20.0	5200	1.25
11	60	10.0	6000	1.15
12	75	10.0	6900	1.14
13	90	5.0	8000	1.10
14	90	7.5	8100	1.16
15	90	10.0	8400	1.12
16	90	20.0	8400	1.34
17	120	7.5	10,400	1.17
18	135	10.0	11,100	1.10
19	135	20.0	11,600	1.37

^a 1^H NMR.

^b^ GPC.

### Self-assembly and crystallization kinetics

To track how PLLA DP influences the self-assembly kinetics, UV/Vis measurements were performed to track the turbidity changes post polymerization for polymers PLLA_45_-*b*-PEG_45_ **9**, PLLA_45_-*b*-PEG_45_ **11**, PLLA_45_-*b*-PEG_45_ **12**, and PLLA_45_-*b*-PEG_45_ **15**, all at 10% solids w/w. (Fig. 1b). Measurements were performed at 600 nm, a wavelength in which no molecular species present in ROPI-CDSA absorb. The data showed that with increasing PLLA DP, the initial rate of self-assembly increases and results in more turbid final solutions.

**Fig. 2:**
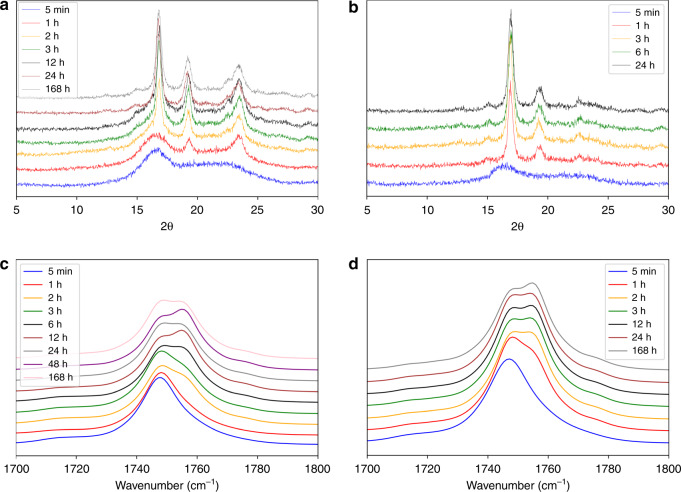
Kinetics of PLLA crystallization. **a**, **b** WAXS pattern for PLLA_45_-*b*-PEG_45_ (10% solids w/w 9) (**a**) and PLLA_90_-*b*-PEG_45_ (10% solids w/w 15) (**b**) over time and **c**, **d** FTIR spectra of the carbonyl shift for 9 (**c**) and 15 (**d**). Note for (**a**), from 24 h to 168 h, no significant difference is observed. For (**b**), from 3 h to 24 h, no significant difference is observed. Note that for (**a**), 5 min and 1 h, and 2b, 5 min show metastable crystalline intermediates. The data show a development in the crystallinity over time indicating that the self-assembly process measured in Fig. 1 is related to the crystallization of the PLLA core-forming block.

To determine if crystallization of the PLLA block is a driving force in the self-assembly, the solutions for PLLA_45_-*b*-PEG_45_(10% solids w/w **9**) and PLLA_90_-*b*-PEG_45_ (10% solids w/w **15**) were freeze-dried at various time points between 5 min and 24 h post polymerization. The dried powders were analyzed by WAXS and FTIR spectroscopy to monitor the crystallization behavior (Fig. 2). Crystallinity is tracked over time by comparing the area of the crystalline peaks to the total area in WAXS patterns (Supplementary Figs. 3–5 and Supplementary Discussion on % crystallinity calculations). In both cases, metastable crystalline intermediates signified by broad peaks were observed at early time points giving way to sharper crystalline peaks at later time points (Fig. 2). For PLLA_45_-*b*-PEG_45_ (10% solids w/w **9**), the PLLA crystallinity increased rapidly during the first three hours to 51% crystalline and then increased slowly, to a maximum of approximately 81% crystalline around 24 h. For PLLA_90_-*b*-PEG_45_ (10% solids w/w **15**), the crystallinity increased at a faster rate, reaching 48% crystallinity within the first h and then increased slowly to 63% at 24 h. The WAXS data is consistent with the development of the α crystalline form of PLLA, which is considered the more thermodynamically stable polymorph (Fig. 2a, b)^49–51^. To compare BCP crystallization to homopolymer crystallization, two PLLA samples with DP = 45 and DP = 90 were crystallized in toluene, which achieved 87 and 86% crystallinity, respectively. This indicates that the presence of the PEG block could inhibit crystallization for longer PLLA blocks. The FTIR spectra show the carbonyl-stretch at 1749 cm^−1^ broadening to form two peaks (1749 cm^−1^ and 1754 cm^−1^), which is also consistent with the crystallization of PLLA (Fig. 2c, d)^49,50,52^. Peak ratios of 1754 cm^−1^ to 1749 cm^−1^ indicate significant changes in the carbonyl environment occurring in the early time points in PLLA_45_-*b*-PEG_45_ (10% solids w/w **9**) and PLLA_90_-*b*-PEG_45_ (10% solids w/w **15**), supporting the WAXS data.

### Self-assembly mechanism

To monitor the morphological evolution, the freeze-dried samples were re-dispersed in water (a non-solvent for PLLA) at 0.5% solids w/w and analyzed by cryo-TEM. Previous examples of CDSA of PLLA-based BCPs in water demonstrate that for self-assembly to occur, solutions must be heated in water (>65 °C) and cooled^20,21^. Thus, at room temperature, we expect that PLLA-*b*-PEG nanoparticles are stable in water. Furthermore, it is well known that crystallinity and nanostructure of PLLA-based BCP are stable to dehydration and resuspension^53^.To confirm that the BCP structures formed in toluene are stable to freeze drying and re-dispersion, several control experiments were performed (Supplementary Figs. 6–11 and Supplementary Discussion on controls). All of these control experiments showed the structures to be stable. Additionally, cryo-TEM analysis of the ROPI-CDSA structures which had been in water several weeks after resuspension, and water resuspensions from dehydrated BCPs aged several months showed stable structures. This indicates that the BCPs form stable kinetically-trapped structures, consistent with previous literature of PLLA-based BCPs in water (Supplementary Fig. 12)^19,20,22^. This also demonstrates that ROPI-CDSA can be used to produce a range of hierarchal PLLA-*b*-PEG structures by kinetically trapping in water at various time points during the self-assembly process.

**Fig. 3:**
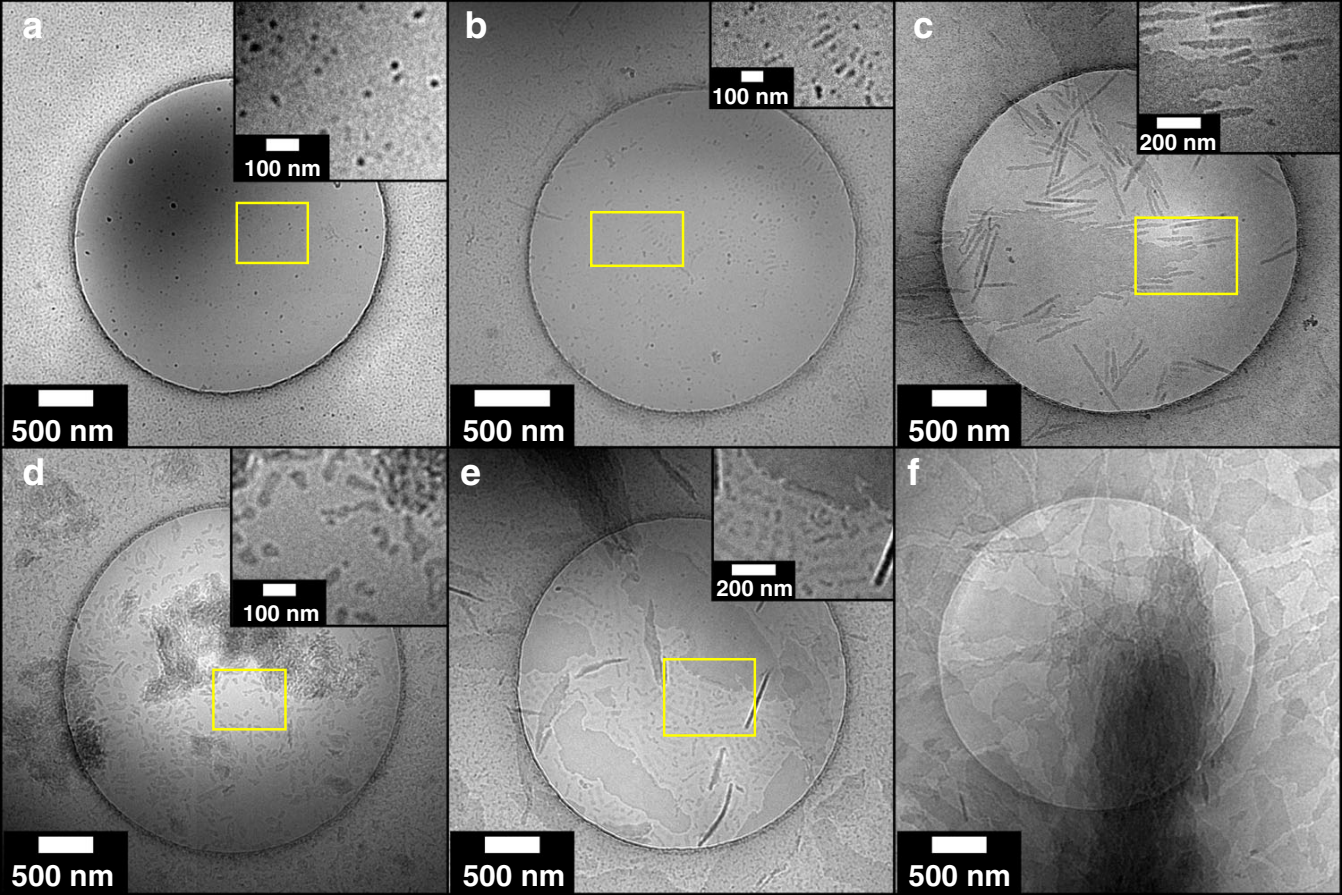
Morphological evolution by cryo-TEM. **a**–**c** PLLA_45_-*b*-PEG_45_ (10% solids w/w 9), **a** = 1 h, **b** = 6 h, **c** = 24 h. **d**–**f** PLLA_90_-*b*-PEG_45_ (10% solids w/w 15), **d** = 5 min, **e** = 3 h, **f** = 6 h. The data show two distinct assembly mechanisms that appear to be a result of unimer addition (sample 9) and particle aggregation (sample 15), resulting in lamellae with different morphologies.

The cryo-TEM data for PLLA_45_-*b*-PEG_45_ (10% solids w/w **9**, Fig. 3a–c) shows that the assembly process proceeds via the formation of spheres (*t* = 1 h diameter = 14.4 ± 2.6 nm Supplementary Fig. 13), that evolve into rods (*t* = 3 h diameter = 15.8 ± 2.3 nm and length = 56.1 ± 42.4 nm Supplementary Figs. 14 and 15), which appear to aggregate and form lamellae (*t* = 24 h thickness = 24.7 ± 2.2 nm length = 3212 ± 927 nm width = 859 ± 308 nm Supplementary Fig. 16). For most time-points, the samples showed a mixture of structures (e.g., spheres and rods or rods and lamellae), which is consistent with a kinetically controlled process^54,55^. On several occasions the rods appear to be aligned (Fig. 3b inset) and in some cases they appear to be connected by long fibers (diameter = 10 nm, length > 1 μm, Supplementary Fig. 17). The distinction between nanorods and nanofibers is typically related to their aspect ratios, where nanorods typically have ratios of 3–15 and fibers typically are >> 15^56^. Here, the fibers appear to act as nucleation sites for the spheres and rods, directing their formation. The cryo-TEM data for sample PLLA_90_-*b*-PEG_45_ (10% solids w/w **15**, Fig. 3d–f) shows a different formation pathway. The initial structures appear to be poorly-defined rod-like precursors (diameter = 29 ± 12 nm length = 90 ± 27 nm Supplementary Fig. 18) which evolve into lamellae (width = 360 ± 250 nm length = 1270 ± 910 nm thickness = 22 ± 2 nm Supplementary Fig. 19) with a second population of rod-like structures (diameter = 22 ± 6 nm length = 101 ± 35 nm Supplementary Fig. 20). After 6 h, the rod-like structures are no longer present, with only lamellae (width = 360 ± 230 nm length = 1300 ± 700 nm thickness = 23 ± 2 nm Supplementary Fig. 21) and 3D lamellae stacks present, confirmed through a tilt series (Supplementary Fig. 22).

### Phase diagram

**Fig. 4:**
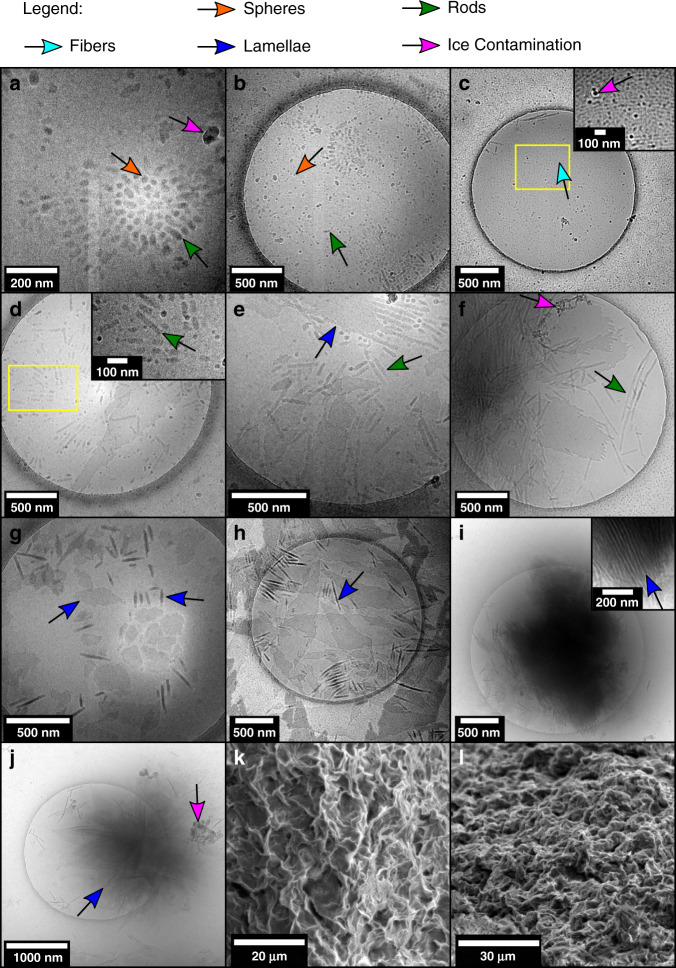
Representative images of ROPI-CDSA morphologies. Selected cryo-TEM (**a**–**j**) and SEM (**k**–**l**) images for representative morphologies of the phase diagram, **a**–**c** is for 1D systems, **d**–**h** are for 2D systems, **i**–**l** are for 3D systems. Images are from the following samples: **a**, **b:** **7**, **c:** **5**, **d**, **e:** **9**, **f:** **6**, **g**, **h:** **13**, **i**: **16**, **j:** **19**, **k**, **l**: **17**. (See Table 1 for a sample guide). The data show that ROPI-CDSA can be used to form block copolymer materials with a wide range of morphologies, dimensions, and length scales.

**Fig. 5:**
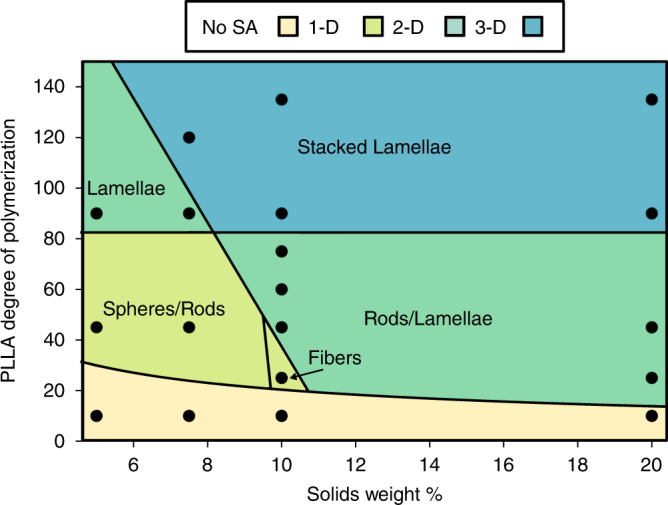
ROPI-CDSA phase diagram. The phase diagram shows hierarchical ordering for samples **1**–**19** (Table 1). No SA refers to no self-assembly observed. The phase diagram shows a clear trend where higher dimensional structures are favored at higher solids content and higher PLLA DPs.

The above data show that the structure of the PLLA-*b*-PEG assemblies varies as a function of both molecular structure and time post polymerization. To construct a phase diagram and verify the reproducibility of ROPI-CDSA^11,14,26^, samples were left for several days post polymerization. However, it is important to note that each sample will likely have its own self-assembly pathway to reach these relaxed, meta-stable structures. Representative cryo-TEM and SEM images of various morphologies are shown in Fig. 4. The samples from Table 1 are plotted as a function of PLLA DP and % solids w/w and constitute a phase diagram shown in Fig. 5. Samples **7** and **8** (PLLA_45_-*b-*PEG_45_ 5% and 7.5% solids w/w, respectively, Fig. 4a, b) formed short rods (width 20–30 nm, length 20–70 nm), sample **5** (PLLA_25_-*b*-PEG_45_ 10% solids w/w) (Fig. 4c) formed fibers (diameter ≈ 10 nm, length > 1 micron) similar to the structure found in Fig. 3b and Supplementary Fig. 17, samples **6** (PLLA_25_-*b*-PEG_45_ 20% solids w/w)**, 9**–**10** (PLLA_45_-*b*-PEG_45_ 10 and 20% solids w/w, respectively)**, 11** (PLLA_60_-*b*-PEG_45_ 10% solids w/w) and (Fig. 4d–f) formed a mixture of rods (width 20–30 nm length 100–1000 nm) and lamellae **12** (PLLA_75_-*b*-PEG_45_ 10% solids w/w) (width 200–1000 nm length 500 nm to 4 μm), and samples **13** (PLLA_90_-*b*-PEG_45_ 5% solids w/w) and **14** (PLLA_90_-*b*-PEG_45_ 7.5% solids w/w) (Fig. 4g, h) formed lamellae (width 100–1000 nm length 200 nm to 3.5 μm). Samples **15**–**16** (PLLA_90_-*b*-PEG_45_ 10 and 20% solids w/w respectively), **17** (PLLA_120_-*b*-PEG_45_ 7.5% solids w/w), and **18**–**19** (PLLA_135_-*b*-PEG_45_ 10 and 20% solids w/w, respectively) (Fig. 4i–l) formed organogels in the toluene solution as confirmed by oscillatory rheology (Supplementary Fig. 23) which indicated that a 3D network formed in solution^57^. Stacks or aggregates of lamellae were observed by cryo-TEM (Fig. 4i, j). Freeze drying of the samples and analyzing the bulk powders by SEM revealed the formation of a 3D porous material. Here individual lamellae are not clearly discernable, although it appears that the materials are likely composed of individual lamellae measuring ~5 μm in length The SEM images are in good agreement with cryo-TEM images collected from the gels, both showing the presence of lamellae and an abundance of 3D stacked lamellae with lengths of 500 nm to 5 μm.

## Discussion

**Fig. 6:**
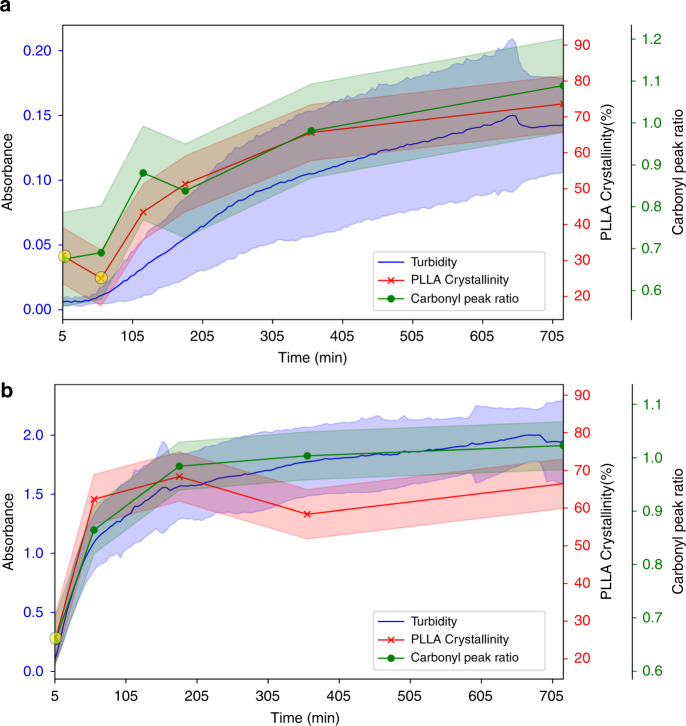
ROPI-CDSA Kinetics. Composite graphs showing the change in turbidity (measured at 600 nm), % crystallinity of PLLA block, and carbonyl peak ratio for **a** PLLA_45_-*b*-PEG_45_ (10% solids w/w **9**) and **b** PLLA_90_-*b*-PEG_45_ (10% solids w/w **15**). The standard error of 3 runs is plotted for turbidity measurements and a calculated error is plotted for crystallinity and carbonyl peak ratio values, see Supplementary information for more details. For the PLLA crystallinity (%) data, early time point metastable crystalline precursors are highlighted with a yellow circle. The data show that the self-assembly kinetics are coincident with the crystallinity kinetics.

**Fig. 7:**
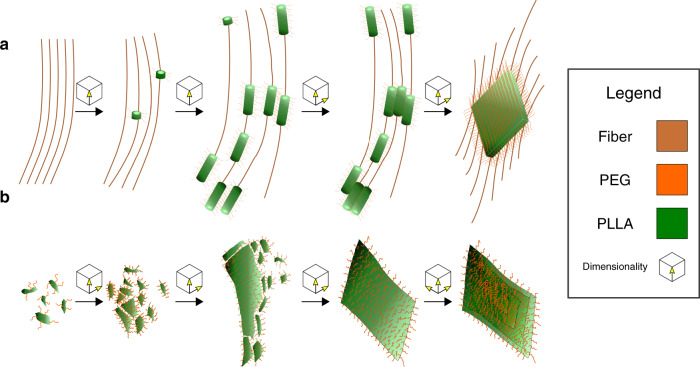
ROPI-CDSA mechanisms. **a** Proposed mechanism for PLLA_45_-*b*-PEG_45_ (10% solids w/w **9**), favoring unimer growth. First, fibers form as a template for 1D growth, giving spheres and rods. Later, these rods aggregate to form 2D lamellae. **b** Proposed mechanism for PLLA_90_-*b*-PEG_45_ (10% solids w/w **15**), favoring aggregation. First, ill-defined 2D rod-like structures form and grow via aggregation. Later, 2D lamellae aggregate, giving 3D lamella stacks.

This work tracks the evolution of PLLA-*b*-PEG morphologies immediately following polymerization, revealing a hierarchical evolution from 0D spheres to 1D rods and fibers to 2D lamellae and, in select cases, to 3D porous networks consisting of stacked or aggregated lamellae. Lamellae are likely favored as polymer crystallinity tends to promote morphologies with low curvature^17,18^, while stacking can minimize surface energies. Comparison of the kinetic data obtained from UV-Vis, WAXS and FTIR shows that crystallization is coincident with self-assembly for both PLLA_45_-*b*-PEG_45_ (10% solids w/w **9**) and PLLA_90_-*b*-PEG_45_ (10% solids w/w **15**), despite the kinetics for both samples being significantly different (Fig. 6). This suggests that the self-assembly is driven by the crystallization of the PLLA block; although, our control experiments show that polymerization is promoted by the presence of the PEG initiator. Consequently, we propose that the assembly process is driven by both the amphiphilicity of the BCP, and the crystallization of the PLLA block^24^. The data showed that the length of the PLLA block strongly influences the self-assembly kinetics and morphology, with longer blocks favoring faster self-assembly (Figs. 1 and 6), and the formation of higher dimensional structures (Fig. 5). This kinetic observation is consistent with previous reports on the CDSA of poly(L-lactide)-*block*-poly(N,N-dimethylacrylamide) (PLLA-*b*-PDMA) diblock copolymers in alcohols, where the kinetic differences were attributed to the difference in solubility of the BCP chain (i.e., more soluble polymers crystallize more slowly)^19,23^. However, the morphological results revealed the more solvophobic PLLA-*b*-PDMA polymers formed lower dimensional structures (1D vs 2D)^19,23^, in contrast to what we report here. For ROPI-CDSA of PLLA-*b*-PEG we see two fundamentally different assembly mechanisms for PLLA_45_-*b*-PEG_45_ (10% solids w/w **9**) and PLLA_90_-*b*-PEG_45_ (10% solids w/w **15**). PLLA_45_-*b*-PEG_45_ (10% solids w/w **9**) (more soluble polymer) appears to initially form high aspect ratio fibers that act as nucleation sights for the crystalline rods, directing the growth to occur along the fiber in one dimension. As rod growth occurs during the time period where most of the crystallinity develops, we propose that these rods form by unimer addition from either the solution or by Ostwald ripening of the fibers^58^. At later time points, these rods aggregate, resulting in 2D growth and the formation of the lamellae (Fig. 7a). PLLA_90_-*b*-PEG_45_ (10% solids w/w **15**) (less soluble polymer) initially appears to form irregularly shaped particles that grow through a particle aggregation mechanism. This results in an initial 2D growth, forming lamellae, and later in 3D growth, forming stacked lamellae and the hierarchical porous network (Fig. 7b). The morphological difference between the lamellae formed in PLLA_45_-*b*-PEG_45_ (10% solids w/w **9**) and PLLA_90_-*b*-PEG_45_ (10% solids w/w **15**) are also consistent with proposed mechanistic differences (Fig. 3). Cryo-TEM images of PLLA_60_-*b*-PEG_45_ (10% solids w/w **11**) and PLLA_75_-*b*-PEG_45_ (10% solids w/w **12**) show morphologies that are consistent with both mechanisms acting simultaneously. The lamellae structures formed by **11** and **12** have characteristics of both **9**, as they are composed of aligned rods, and **15**, in that the lamellae are irregularly shaped (Supplementary Fig. 24).

These two different initial growth mechanisms (templated unimer addition and particle aggregation) are consistent with growth models previously reported for small molecules and inorganic crystals^58,59^, where the observed differences in formation pathways are explained by considering the relative energy barriers for either pathway. In our work, the longer PLLA block reduces the energy barrier for crystallization and raises the energy barrier for unimer exchange. However, one important consideration for macromolecular self-assembly that is not present for small molecules or inorganic crystals is the influence of chain stretching.

**Fig. 8:**
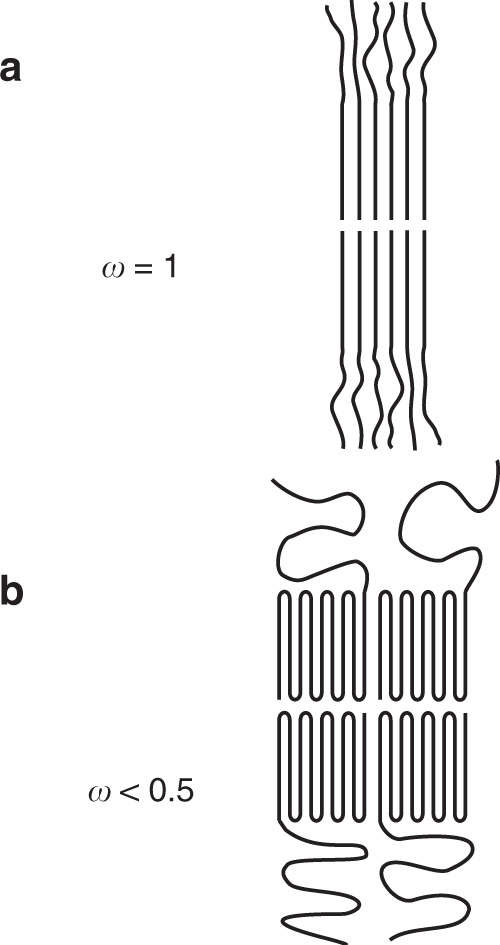
Polymer chain stretching. **a** When *ω* = 1 giving a dense corona and **b** when *ω* < 0.5 giving significant coiling. In ROPI-CDSA, PLLA_45_-*b*-PEG_45_ (10% solids w/w **9**) has a denser corona, in-between *ω* = 1 and *ω* < 0.5. In contrast, PLLA_90_-*b*-PEG_45_ (10% solids w/w **15**) has a *ω* < 0.5 exhibiting more coiling.

The dimensions of self-assembled BCP structures are determined by the aggregation number and the stretching or coiling of the polymer chains^17,60^. For spheres, rods, and lamellae, the degree of polymer chain stretching (*ω*) can be determined by comparing the measured radius (*r*) (or half the lamella thickness) to the maximum length of the polymer chain (*L*_max_) (Fig. 8) as shown in Eq. (1).

1 $$\omega = \frac{{\it{r}}}{{{\it{L}}_{{\rm{max}}}}}$$

From distance calculations using CrystalMaker® software, the molecular length of a PLLA monomer is 3.69 Å Supplementary Fig. 25 and Supplementary Methods). The total fully extended PLLA backbone lengths (*L*_max_) are 16.6 nm and 33.2 nm for PLLA_45_-*b*-PEG_45_ (10% solids w/w **9**) and PLLA_90_-*b*-PEG_45_ (10% solids w/w **15**), respectively. This would result in a theoretical maximum fiber/rod diameter and/or lamella thickness of 33.2 nm for PLLA_45_-*b*-PEG_45_ and 66.4 nm for PLLA_90_-*b*-PEG_45_. For **9**, the fibers have an ω of 0.33 (0.29–0.37); for the rods and lamella, *ω* changes over time from *ω*_1hr_ = 0.42 (0.34–0.50) to *ω*_24 hr_ = 0.74 (0.68–0.81), becoming more stretched out during the self-assembly process. (N.B. lamella interdigitation is ruled out as we observed *ω* > 0.5). For **15**, *ω* stays relatively constant, at about 0.35 (0.32–0.37). In comparison to PLLA_45_-*b*-PEG_45_ (10% solids w/w **9**), PLLA_90_-*b*-PEG_45_ (10% solids w/w **15**) has a longer PLLA block and a smaller *ω*. This results in particles of **15** having a substantially lower corona density than **9**. We propose that this promotes particle aggregation events by lowering the barrier to inter-lamellar core-core interactions^61^. We propose that this also facilitates stacking and the formation of structures in 3D.

**Fig. 9:**
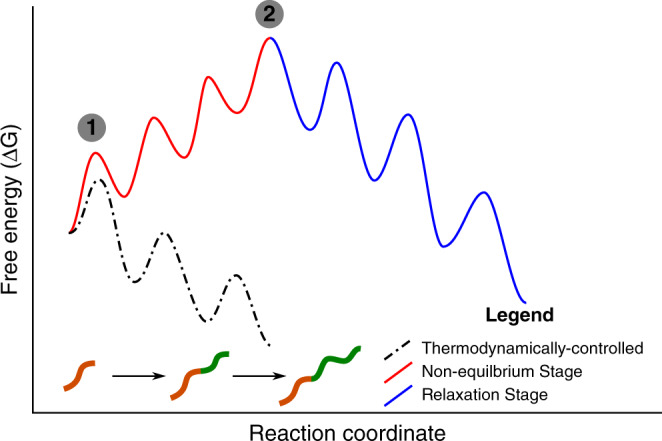
Free energy diagram for polymerization-induced self-assembly. The dashed lines represent the situation where the relaxation time is short compared to the polymerization time. Consequently, with each monomer addition, the growing block copolymer relaxes to a lower energy configuration. The result is that the reaction proceeds downhill and finishes when the polymerization terminates. The solid line represents the situation where the polymerization time is much shorter than the relaxation time. Consequently, with each monomer addition, the growing block copolymer cannot fully relax to a lower energy configuration. The result is that the reaction initially proceeds uphill (Stage 1) and then relaxes post polymerization (Stage 2). In Stage 2, the energy barriers represent morphological transitions (e.g., spheres to rods or rods to lamellae).

An interesting feature of PISA experiments is that the molecular structure of the species assembling changes during the assembly process. As it is well known that the thermodynamics and kinetics of self-assembly change with molecular structure^8,62,63^, PISA processes occur as a result of the evolving energy landscape. As the initial homopolymer and monomer are soluble, the system is initially ‘in-equilibrium’ (and dissolved). Every addition of monomer to the end of the homopolymer chain creates a building block (the growing block copolymer) that is higher in free energy (due to its increasingly amphiphilic nature). The majority of PISA experiments can be categorized as either thermodynamically-controlled^14,26,64^ or kinetically-trapped^2,26,27,34,64–66^, which differ based on the type of evolving energy landscape. In the thermodynamically controlled systems, the energy landscape is relatively smooth compared to *k*_B_*T*, resulting in very short relaxation times. With each monomer addition, the landscape changes and the system relaxes to its thermodynamic minimum. Consequently, the system evolves down a thermodynamic pathway and the design of these systems is based on thermodynamic considerations^28^. In the kinetically-trapped systems, the landscape evolves such that it becomes rough compared to *k*_B_*T*, at which point the system gets trapped in its configuration^29,34^. In this system, the polymer evolves thermodynamically until the morphology gets locked in place by the changing energy landscape. The key feature of both these types of PISA processes (thermodynamically and kinetically controlled) is that the relaxation time is much faster than the polymerization time^67^. Consequently, after each monomer addition, the system relaxes to a lower energy state and the overall reaction coordinate proceeds downhill (Fig. 9) and the assembly is finished once the polymerization finishes. In the system described here, the roughness of the energy landscape is on the order of *k*_B_*T*. The consequence of this is that the relaxation times (driven by the crystallization kinetics) are long (hours to days). As the polymerization times are short (seconds to minutes), this creates a situation where with each monomer addition, the system becomes increasingly further from its minimum free energy organization as the system does not have time to relax to a lower energy state (Fig. 9). Therefore, we propose that during ring-opening polymerization, it is the release of the lactide ring strain, which drives a non-equilibrium self-assembly evolution (See Supplementary Discussion on non-equilibrium assembly)^55,68^. Although not all of the chemical energy stored in the monomer will be converted into free energy to drive self-assembly (some being lost as heat energy), as the growing polymer chain is unstable, it has a higher free energy than the soluble homopolymer. Interestingly, the ring strain release during lactide polymerization (−23 kJ/mol)^69^, is similar to the energy stored in GDP-rich microtubules, a classic out-of-equilibrium biological system (−22 kJ/mol)^70,71^. Microtubule assembly is a classic dissipative assembly process. The dissipative nature of the assembly is related to the reversibility of the chemistry, which is common in out-of-equilibrium systems^71–73^. In the presented PLLA-*b*-PEG systems, while the self-assembly is initiated through a modification of the molecular structure, the chemistry is non-reversible, which is advantageous for using the process to trap the meta-stable structures. Here, this is achieved by re-dispersing the structures in water, where the energy landscape (at room temperature) is rough compared to *k*_B_*T*. Consequently, we can divide the process into two stages. Stage 1 is the non-equilibrium assembly process that occurs during polymerization (Fig. 9, stage 1). Stage 2 is the relaxation process that occurs as the high energy structures relax to a lower energy configuration (Fig. 9, stage 2). In this paper, we have only investigated the relaxation process; however, our control experiments show that the presence of the PEG block is essential for the polymerization to occur efficiently, indicating that some assembly is taking place during stage 1. This is justified from the evidence that kinetically-trapped BCPs structures, are highly dependent on the assembly mechanisms^61,74–76^. Furthermore, comparing kinetically-trapped structures in water using the same BCP but different methods (solvent switch and CDSA, see also Supplementary Figs. 7–10) shows that ROPI-CDSA forms by a different pathway. Our hypothesis is that this pathway is dependent on the non-equilibrium stage of the assembly process (Fig. 9, stage 1), and can provide access to meta-stable precursors that do not occur during the other assembly methods. Our data show two mechanisms by which meta-stable precursors can direct self-assembly. First, the formation of the highly coiled amorphous fibers which directs the location and dimensionality of the crystallization process. Second, the formation of precursor particles with low corona densities which facilitates 2D and 3D growth through particle aggregation. Although more work is needed to investigate the non-equilibrium assembly process, the prospect of controlling PISA assembly mechanisms by changing the relative rates of polymerization and self-assembly provides an exciting opportunity to create polymeric materials based on kinetic considerations (rather than thermodynamic considerations). For example, Khor et al.^77^. demonstrated that changing the rate of polymerization in emulsion RAFT PISA experiments resulted different morphological outcomes. Specifically, that a faster rate of polymerization provides access to polymer vesicles instead of spheres (achieved at slower rates of polymerization). Although not discussed in the text, a plausible explanation for this is that the faster rate of polymerization provides access to a non-equilibrium state that enables relaxation to vesicles. This study is an important example which, in addition to the work presented here, highlights that further study of the non-equilibrium behavior in PISA experiment can provide access to unique assembly processes and potentially to unique structures.

In conclusion, ROP of L-lactide in toluene, using a monofunctionalized PEG initiator, and TBD as a catalyst, results in the polymerization-induced crystallization-driven self-assembly of PLLA-*b*-PEG block copolymers. This method, termed ROPI-CDSA, can produce 1D, 2D, and 3D structures with concentrations ranging from 5 to 20% solids w/w. As the rates of the polymerization are faster than the rates of self-assembly and crystallization, the initial structures generated post polymerization are very far from equilibrium. Over time, driven by crystallization and solvent compatibility, the system relaxes to a lower energy configuration. The hierarchical growth is controlled by three processes operating on different time-scales, polymerization, amphiphilic self-assembly, and crystallization. Polymerization creates an amphiphile which can self-assemble by microphase separation, and a polymer capable of undergoing crystallization-driven self-assembly. The interplay between these three processes is complex but provides a rich design space to create block hierarchical copolymer materials. The growth mechanism can occur via a predominantly unimer addition or particle aggregation mechanism. Understanding of the mechanism and kinetics of the relaxation process allows a range of meta-stable structures to be trapped by freeze drying, from a single BCP. The freeze-dried structures can then be stored and redispersed in water as required. This work provides a basis for the use of ROPI-CDSA to generate hierarchical 1D, 2D, and 3D materials using biocompatible and biodegradable polymers with a scalable one pot strategy. It additionally outlines an approach for utilizing PISA as a method to create non-equilibrium self-assembly processes and trap unique meta-stable structures.

## Methods

### Materials

mPEG_45_ (MW = 2000) (Sigma Aldrich) was azeotropically distilled ×2 in toluene and high-vacuumed overnight. L-lactide (TCI) was recrystallized in toluene ×3. Anhydrous toluene (99.8%), and triazabicyclodecene (TBD) were purchased from Sigma Aldrich and were used without further purification. Benzoic acid (Fisher Chemical) was used without further purification. Milli-Q water (ρ > 18 MΩ cm) was used as the solvent for all aqueous solutions. Chemicals were stored in a dry-N_2_ atmosphere glove box. Reactions were performed in a N_2_ glove box.

### PLLA-*b*-PEG synthesis and self-assembly

Procedure adapted from Waymouth et al.^43^ L-lactide (64.9 mg, 0.45 mmol, PLLA target DP = 45) was added to a solution of mPEG_45_ (40 mg, 20 μmol) in 1.08 mL of toluene (10% solids w/w). 15 μL (0.1% mol) TBD from a toluene stock solution (4.3 mg/mL) was then added. The solution was stirred for 90 s and subsequently quenched with 0.05 mL benzoic acid stock solution (100 mg/mL). We noticed that the rate of stirring influenced the self-assembly kinetics and therefore stirring was kept at 400 rpm for reproducibility (For full results see also Supplementary Table 1, for additional synthetic information see Supplementary Table 2). ^1^H NMR (500 MHz, CDCl_3_) δ 5.16 (q, *J* = 7.0 Hz, CH, PLLA backbone), 5.03 (q, *J* = 6.7 Hz, CH L-lactide), 3.72–3.59 (m, CH_2_ PEG backbone), 3.54 (dd, *J* = 5.6, 3.6 Hz, CH_2_, PEG), 3.37 (s, 3H, terminal CH_3_ PEG), 1.67 (dd, *J* = 6.7, 1.5 Hz, CH_3_ L-lactide), 1.58 (d, *J* = 6.7 Hz, CH_3_ PLLA backbone), 1.50 (dd, *J* = 14.7, 7.0 Hz, terminal CH_3_ PLLA) (see also Supplementary Fig. 1).

### Preparation methods of aqueous solutions

Lyophilized powders were obtained by freezing the toluene solutions in a round bottom flask with liquid nitrogen followed by sublimation using a vacuum pump. Resuspension of the powders was aided by sonication for 30 min in a Branson 3800 Ultrasonic Cleaner. Solvent extractions were performed by dropping a few (5–10) μL of toluene solution into excess water and vortexing for 10 s. In both cases aqueous solutions with a concentration of 0.5 mg/mL (0.5% solids w/w) were obtained and cryo-TEM analysis showed similar results for both preparations.

### Structural characterization

Proton nuclear magnetic resonance (^1^H NMR) spectra were collected on a 500 MHz Bruker Avance spectrometer in CDCl_3_. Chemical shifts are given in ppm, calibrated from residual CHCl_3_. Gel permeation chromatography (GPC) was performed in DMF using an Agilent 1100 chromatograph equipped with RID detector and a PL gel 5 μm 300 × 7.5 mm mixed column. Samples were calibrated against polystyrene standards (see also Supplementary Fig. 2 for GPC traces).

### Turbidity measurements

Self-assembly kinetics were measured with UV/Vis spectroscopy on a Thermo Scientific NanoDrop 2000c. Changes in turbidity were measured at 600 nm every 15 s for 720 min with a moderate stirring rate. Triplicate runs were taken of each sample (Supplementary Fig. 26). Plots shown in paper are binned by a factor of 20.

### Crystallization measurements

Wide-angle X-ray scattering (WAXS) patterns were measured on a Rigaku Smart lab X-ray diffractometer in Bragg-Brentano diffraction mode utilizing X-rays generated at 40 kV and 44 mA with Cu Kα irradiation (step size 0.2 deg, speed 1.0, IS 2/3 deg, RS1 2/3 deg, RS2 0.3 mm). Approximately 20 mg of a lyophilized sample was used in measurements. PLLA crystallinity was originally calculated using Smart Lab (Rigaku) software (see also Supplementary Fig. 4 and Supplementary Tables 4 and 5), but due to the possibility of overlapping PEG peaks, a deconvolution routine was employed to separate PEG and PLLA peaks (see also Supplementary Fig. 5 and Supplementary Tables 6 and 7). Using Smart Lab software, following background correction, amorphous peak and crystalline peak areas were defined using the previous peak assignment of PLLA-*b*-PEG^51,78^. Additional crystallinity analysis was conducted using a custom peak fitting python script. The area of interest was restricted between 10 and 30° and a constant background was subtracted. After which a model function was created as the sum of a mixture of Voigt and Gaussian components. The model was subsequently fitted to the experimental data by optimizing the individual component parameters (peak center, amplitude, sigma, and gamma). The quality of the fit was assessed by measuring the mean square error between the model and the experimental data. The parameters and script used to fit the data is discussed further in the SI. In both cases, crystallinity was calculated from the area of crystalline peaks as a percentage of the total peak area (For more details on peak assignment see SI). The percentage of crystallinity was normalized by the mass ratio of the PLLA segment. Fourier transform infrared (FTIR) absorbance spectra were collected on a Jasco 4700 FTIR from lyophilized samples (Supplementary Figs. 27 and 28).

### Cryogenic-transmission electron microscopy (cryo-TEM)

Cryo-TEM samples were prepared from resuspended or extracted solutions onto Quantifoil R2/2 (Electron Microscopy Sciences) grids. Grids were glow discharged for 70 s to increase hydrophilicity prior to sample loading. Vitrification was carried out by an Automatic Plunge Freezer ME GP2 (Leica Microsystems) with 3 μL of sample. Grid preparation was performed at 95% humidity and the grids were blotted for 3 s prior to plunging into liquid propane. Cryo-TEM samples were then placed on a Gatan Cryo-TEM holder and imaged on a JEOL 2100F TEM using a Schottky type field emission gun operating at 200 keV. Images were recorded using DigitalMicrograph (Gatan) software with a Gatan OneView CMOS camera at 4k × 4k resolution.

### Scanning electron microscopy (SEM)

Samples were prepared from lyophilized samples which were freeze-cracked in liquid N_2_ and coated ex-situ with 3 nm of iridium (Quorum, Q150T Plus). Secondary electron images were collected on a FEI, Quanta 3D FEG with Everhart-Thornley detector, using a 5 kV acceleration potential, and a probe current of 200 pA.

## Supplementary information

Supplementary Information

## Data Availability

The datasets generated during and/or analyzed during the current study are available from the corresponding author on reasonable request.

## Code availability

The code for deconvoluting the WAXS data is available from the corresponding author on reasonable request.
