# Supplementary material for: Ring-opening polymerization-induced crystallization-driven self-assembly of poly-L-lactide-block-polyethylene glycol block copolymers (ROPI-CDSA)
Source: Nat Commun. 2020 Sep 17;11:4690. doi: 10.1038/s41467-020-18460-2 (PMC7499262; doi:10.1038/s41467-020-18460-2)
Supplement: Supplementary file 1 — Supplementary Information [file 41467_2020_18460_MOESM1_ESM.pdf]

Supplementary information for Ring-opening polymerization-induced crystallization-driven self-assembly of poly-L-lactide-*block*-polyethylene glycol block copolymers (ROPI-CDSA)

Paul J. Hurst[1], Alexander M. Rakowski[1], Joseph P. Patterson[1]*

[1]*Department of Chemistry, University of California, Irvine, Irvine, CA 92697-2025,*

Corresponding author email: patters3@uci.edu

SUPPLEMENTARY DISCUSSION

% Crystallinity Calculations: Crystallinity is tracked over time by comparing the area of the crystalline peaks to the total area in WAXS patterns. The data shows that the crystallinity is predominantly from the PLLA block, however we noted additional peaks in diffraction pattern of samples with PLLA DP 25 and 45 which corresponds to PEG crystallinity. An analysis of WAXS patterns of PLLA-*b*-PEG with increasing DP of PLLA at 20% solids w/w also show PEG crystallinity present for $PLLA_{25}$-*b*-PEG_{45} **6** and $PLLA_{45}$-*b*-PEG_{45} **10** but absent for $PLLA_{90}$-*b*-PEG_{45} **16** and $PLLA_{135}$-*b*-PEG_{45} (Supplementary Fig. 5). It is known that in bulk BCP crystallization of PLLA-*b*-PEG, lower DP PLLA BCPs exhibit marked PEG crystallinity.[1] Thus, we split the PLLA peaks from PEG peaks using previous polymer crystallization data (Supplementary Fig. 4, Tables S6-7, Supplementary Methods)[2, 3] For the PLLA 90 samples the kinetic study shows that the PEG crystallinity is relatively constant over time (unlike the PLLA crystallinity which increases overtime) and therefore we do not believe the PEG crystallinity is a significant driving force of the self-assembly. We also note that both models (convoluted and unconvoluted peaks) discussed in the methods section show the same general trend with respect to the differences between the PLLA DP 45 and 90 samples (Tables S4-7, Supplementary Methods).

Controls: A control experiment was performed by re-dispersing poly-L-lactide-*block*-polyethylene glycol (PLLA-*b*-PEG) block copolymer (BCP) into water that has been synthesized in dichloromethane, where no self-assembly take place (Supplementary Fig. 6). The control BCP failed to disperse in water whereas the ROPI-CDSA polymers readily formed turbid solutions. A second control was performed by preparing PLLA-*b*-PEG

through a solvent switch process (tetrahydrofuran to water) and a CDSA process (heating to 65 °C and cooling), in both cases the structure formed are significantly different to those generated by ROPI-CDSA (Supplementary Fig. 7-10). This shows that the structures formed during the ROPI-CDSA processes are essential for creating the assemblies in water. Preparation of the cryogenic-transmission electron microscopy (cryo-TEM) samples directly in toluene was challenging due to partial/full evaporation of the toluene during the blotting stages, however comparison of the same samples prepared directly in toluene and by freeze drying showed similar structures (Supplementary Fig. 11).

Dispersion vs. emulsion PISA: PISA processes can be categorized as dispersion PISA, where both the monomer and stabilizer block are soluble in the reaction solvent or emulsion PISA, where the monomer is stabilized by an emulsifier.[4] Here, L-lactide monomer has a limited solubility in toluene of about 60 mg/mL. Consequently, in solutions with lower target DP or lower solids content, ROPI-CDSA is a dispersion PISA process, However, in solutions with higher target DP and/or high % solids w/w, not all L-lactide dissolves in toluene (even with long stir times). Upon the beginning of the polymerization, the excess L-lactide dissolves in solution because of decreasing L-lactide concentration. Following polymerization, the solutions remain clear until the onset of turbidity. In these experiments ROPI-CDSA has features of both dispersion and emulsion PISA. In addition, from low conversion of homopolymerization in toluene, we know the PEG stabilizer block is necessary to enable the polymerization in toluene. This observation is analogous with the ability of the macroinitiator increasing the polymerization rate constant (k_p) as the growing block becomes more insoluble.

Non-equilibrium PISA: The term "non-equilibrium" is often used interchangeably with "far-from-equilibrium" assemblies.[5, 6] Mattia and Otto,[5] have provided clear descriptions of the following important terms which we quote from their paper:

"Equilibrium assemblies. Systems that are likely to persist for a long time due to their thermodynamic stability.

Kinetically trapped assemblies. Systems that are transiently durable, trapped in a local minimum of the energy landscape. It would take time, or activation energy, for them to be converted to more stable structures.

Far-from-equilibrium assemblies. Systems that require a continuous supply of energy to persist. If the energy supply stops, the system would fall apart and end up in a thermodynamic minimum state (or in a kinetic trap en route). The continuous energy-driven transformation that these structures undergo makes it possible for them to have interesting, and sometimes unpredictable, emergent functions."

Here, we refer to "equilibrium assemblies" as being "thermodynamically controlled" processes because for polymeric assemblies it is almost impossible to determine if they are really in-equilibrium. But it is possible to provide evidence they are thermodynamically controlled, for example, by assembling them under different conditions to see if they form the same structures.[7]

It is clear that in any PISA process there is some non-equilibrium behavior as PISA inherently creates an unstable building block (the growing amphiphile) from a stable building block (the soluble homopolymer). However, we believe it is only useful to apply the term non-equilibrium if there is some practical advantage to the creation of this non-

equilibrium state. In situations where the structural evolution of the assemblies is much faster than the polymerization time,[8-11] the non-equilibrium state that occurs after the monomer is added (but before the system reorganizes to a new structure) is so transient that it is not practically useful, or at least, there have been no demonstrations that it is practically useful. Consequently, we refer to these situations as being thermodynamically controlled because the structures at each stage can be predicted based on the thermodynamics of the polymer structures and its environment. In this regime, changing the polymerization kinetics will only change the rate at which the structures evolve but it will not provide access to different structures. For PISA processes that result in kinetic trapping it is important to note that if the evolution of the assemblies is much faster than the polymerization time then the formed structures are still based on thermodynamics, because the result is a trapped structure that was formed by an 'in-equilibrium' assembly process. In this paper, the practical advantage comes from the ability to trap multiple different structures as they evolve in solution after the polymerization has finished.

SUPPLEMENTARY METHODS

Homopolymer Synthesis: Homopolymer synthesis was carried out following the procedure in the text apart from exchanging mPEG for ethanol. Samples were synthesized in both toluene and dichloromethane.

% PLLA Crystallinity Calculations

To calculate %PLLA crystallinity values the area of the discernable crystalline peaks was divided by the total area subtracted from the background as in Supplementary Equation 1:

$$\% \, Crystallinity = \frac{Area \, Under \, Crystalline \, Peaks}{Total \, Area \, under \, Peaks - Background} * 100\% \quad (1)$$

As preparing WAXS samples was time-intensive, several triplicate of one sample **15** ($PLLA_{90}$-*b*-PEG_{45} 10% solids w/w 24 hours) were used establish a baseline error of ± 5.0%. FTIR peak ratio error was also established by the same method giving a baseline error of ± 0.045.

PLLA Crystallinity was calculated by dividing crystallinity by the % wt. PLLA of PLLA-*b*-PEG block copolymers as in Supplementary Equation 2:

$$\% \, PLLA \, Crystallinity = \frac{\% \, Crystallinity}{\% \, wt \, PLLA} * 100\% \quad (2)$$

% wt. PLLA for $PLLA_{45}$-*b*-PEG_{45} (10% solids w/w **9**) and $PLLA_{90}$-*b*-PEG_{45} (10% solids w/w **15**) is 62% and 76% respectively.

Control Self-Assembly Experiments: All controls were synthesized using TBD in dichloromethane (rather than toluene) where no self-assembly or crystallization should take place. Controls were also purified by precipitation in diethyl ether.

Resuspension in water: Amorphous PLLA-*b*-PEG and Milli-Q water (ρ> 18 MΩ cm) were mixed to give a concentration of 0.5 mg/mL. The resulting suspension was sonicated for 30 minutes. The suspension did not dissolve as indicated visually in Supplementary Figure 6.

Self-assembly by solvent switch: PLLA-*b*-PEG was dissolved in THF to give a concentration of 10mg/mL. Milli-Q water was added in fractions a tenth of the THF added. Fractions were spaced twenty minutes apart while the sample stirred. Once samples had

reached turbidity, no more additional water was added, and samples were dialyzed three times overnight to remove residual THF.

<u>*CDSA in water*</u>*:* PLLA-*b*-PEG and Milli-Q water were mixed and heated to 65 °C. Water was added until the polymer fully dissolved. The solution was heated at 65 °C while stirring and was rapidly cooled to room temperature.

<u>*CDSA in toluene:*</u> PLLA-*b*-PEG was mixed with toluene to give similar concentrations as in ROPI-CDSA. Solutions were stirred for one day and inspected visually for signs of gelation (e.g. how viscous the solution was on upon inverting the vial).

Organic cryo-TEM grid preparation: Cryo-TEM samples from toluene were prepared from Quantifoil grids or graphene oxide coated lacey carbon grids (Electron Microscopy Sciences). Samples were diluted with toluene to give a concentration of 0.5 mg/mL right before sample preparation. Vitrification was attempted by an Automatic Plunge Freezer ME GP2 (Leica Microsystems) with 3 μL of sample. Sample preparation was carried without utilization of a humidity chamber and blotted for 1 s before auto-plunging into liquid nitrogen. Blot times longer than 1 s resulted in grids too thick to image caused by toluene evaporation.

Measurements of Cryo-TEM images: Cryo-TEM images were measured using DigitalMicrograph (Gatan) software. Line profiles with integrations of 50 pixels were made to measure width accurately. Length was measured from structure end to end. Lamella width and length were measured from the widest and longest points respectively. Lamella thickness was determined by measuring lamellae perpendicular to the image plane (For

an example of this, see Figure 4i). About 10-50 measurements were made on each sample.

Rheology: Oscillatory rheology was collected from organogels on a TA DHR 2 rheometer. Gels were loaded using a 20 mm steel Peltier plate. Measurements were taken from $1.0e^{-3}$ to 100.0 strain % at 25 °C.

PLLA Length Calculation: Using CrystalMaker®, a short PLLA chain was simulated and allowed to relax to a minimum energy configuration. After which the distance was measured between one PLLA unit as shown in Supplementary Figure 24.

SUPPLEMENTARY TABLES

Supplementary Table 1: Synthesis and Structural Characterization of PLLA-*b*-PEG in ROPI-CDSA for experimental information see the methods section and Supplementary Table 2.

**Reference**	**[M]/[I]**	**DP**	**% Conv.**	**% solids w/w**	**NMR M_n**	**GPC M_n**	**M_w**	**Đ**	**Structure***
**1**	5	10	90.1	5.0	2700	10820	11540	1.066	N
**2**	5	10	91.3	7.5	2700	10600	11340	1.070	N
**3**	5	10	92.6	10.0	2700	10960	11810	1.078	N
**4**	5	10	97.0	20.0	2700	10103	11990	1.089	N
**5**	12.5	25	97.1	10.0	3700	13990	15460	1.105	S/R
**6**	12.5	25	97.1	20.0	3700	13320	15260	1.145	L/R
**7**	22.5	45	93.8	5.0	5200	17230	19360	1.124	S/R
**8**	22.5	45	94.3	7.5	5400	19620	21930	1.118	S/R
**9**	22.5	45	96.7	10.0	5300	17810	20570	1.155	L/R
**10**	22.5	45	94.0	20.0	5200	18840	23480	1.246	L/R
**11**	30	60	93.3	10.0	6000	18830	21740	1.154	L/R
**12**	37.5	75	92.1	10.0	6900	22020	25130	1.141	L/R
**13**	45	90	94.5	5.0	8000	26010	28660	1.102	L
**14**	45	90	96.2	7.5	8100	26970	31140	1.155	L
**15**	45	90	96.2	10.0	8400	25770	30900	1.199	SL/L
**16**	45	90	99.0	20.0	8400	23370	31670	1.355	SL/L
**17**	60	120	96.8	7.5	10400	30150	35140	1.165	SL/L
**18**	67.5	135	93.5	10.0	11100	31980	35160	1.099	SL/L
**19**	67.5	135	99.0	20.0	11600	27030	37130	1.374	SL/L

* Structure legend: SL=stacked lamella L=lamella, R=rods, S=spheres, N=no self-assembly

Note that GPC results are calibrated to PS standards and don’t accurately reflect the molar mass of PLLA-*b*-PEG.

Supplementary Table 2: Experimental Setup for synthesis of PLLA-*b*-PEG in ROPI-CDSA. For experimental information see the methods section. For the structural outcome of the synthesis of PLLA-*b*-PEG see Supplementary Table 1.

Reference	[M]/[I]	Target DP	L-lactide (mg)	L-lactice (mmol)	% solids w/w	Toluene (mL)	TBD stock (μL)	% Conv.
**1**	5	10	14.4	0.10	5.0	1.19	4	90.1
**2**	5	10	14.4	0.10	7.5	0.77	4	91.3
**3**	5	10	14.4	0.10	10.0	0.56	4	92.6
**4**	5	10	14.4	0.10	20.0	0.25	4	97.0
**5**	12.5	25	36.0	0.25	10.0	0.79	8	97.1
**6**	12.5	25	36.0	0.25	20.0	0.35	8	97.1
**7**	22.5	45	64.9	0.45	5.0	2.29	15	93.8
**8**	22.5	45	64.9	0.45	7.5	1.49	15	94.3
**9**	22.5	45	64.9	0.45	10.0	1.08	15	96.7
**10**	22.5	45	64.9	0.45	20.0	0.49	15	94.0
**11**	30	60	86.5	0.60	10.0	1.31	20	93.3
**12**	37.5	75	108.1	0.75	10.0	1.53	25	92.1
**13**	45	90	129.7	0.90	5.0	3.71	30	94.5
**14**	45	90	129.7	0.90	7.5	2.42	30	96.2
**15**	45	90	129.7	0.90	10.0	1.75	30	96.2
**16**	45	90	129.7	0.90	20.0	0.78	30	99.0
**17**	60	120	173.0	1.20	7.5	3.03	39	96.8
**18**	67.5	135	194.6	1.35	10.0	2.43	44	93.5
**19**	67.5	135	194.6	1.35	20.0	1.08	44	99.0

Note that all polymerizations use 40 mg (20 μmmol) of $mPEG_{45}$ and 0.1% molar to L-lactide of TBD. The TBD stock solution has a concentration of 4.3 mg/mL

Supplementary Table 3: Homopolymer Synthesis Results

Target DP	Solvent	% Conversion	NMR M_n
**45**	Toluene	22.5	780
**90**	Toluene	28.6	1900
**45**	Dichloromethane	96.9	3200
**90**	Dichloromethane	97.9	6400

Supplementary Table 4: Unconvoluted WAXS Data for **9**: $PLLA_{45}$-*b*-PEG_{45} 10% solids w/w. Units are degrees unless otherwise stated. Note that the WAXS experimental parameters are given in the main text whereas the crystallinity calculations are given in the supplementary information.

**Time point**	**1 0 3**	**0 1 0**	**1 1 0 / 2 0 1**	**2 0 3**	**Amorphous**	**2 1 0**	**2 1 6**	**PEG**	**Crystallinity (%)**	**PLLA Cryst. (%)**
**5min**	-	-	16.37	-	21.92	-	-	-	10.3	16.7
**1hr**	-	16.15	16.911	19.283	21.4	-	-	23.46	21.4	34.6
**2hr**	12.03	15.28	16.815	19.272	22.46	22.51	-	23.42	28.8	46.6
**3hr**	12.61	15.04	16.821	19.255	20.84	22.84	29.06	23.43	36.3	58.7
**6hr**	12.49	16.22	16.808	19.242	21.03	22.48	29.33	23.44	40.30	65.2
**12hr**	13.8	15.92	16.774	19.248	21.33	22.59	29.27	23.34	43.8	70.8
**24hr**	12.73	14.88	16.75	19.180	20.43	22.46	29.19	23.43	54.26	87.3

Supplementary Table 5: Unconvoluted WAXS Data for **15**: $PLLA_{90}$-*b*-PEG_{45} 10% solids w/w. Units are degrees unless otherwise stated. Note that the WAXS experimental parameters are given in the main text whereas the crystallinity calculations are given in the supplementary information.

**Time point**	**1 0 3**	**0 1 0**	**1 1 0 / 2 0 1**	**2 0 3**	**Amorphous**	**2 1 0**	**2 1 6**	**Crystallinity (%)**	**PLLA Cryst. (%)**
**5min**	-	-	16.504	-	25.23	-	-	15.11	19.8
**1hr**	12.86	14.90	16.877	19.31	20.85	22.57	29.22	50.10	65.6
**3hr**	12.81	15.04	16.847	19.24	19.55	22.56	29.20	54.9	71.9
**6hr**	12.73	15.17	16.946	19.30	19.07	22.82	29.46	57.14	74.7
**24hr**	12.91	15.05	16.905	19.29	21.9	22.61	29.26	59.10	77.4

Supplementary Table 6: Convoluted WAXS data with Voigt/Gaussian model for **9**: $PLLA_{45}$-*b*-PEG_{45} 10% solids w/w. Units are degrees unless otherwise stated. Peaks are color coded with the following: PLLA-green, PEG-yellow, amorphous-blue. Amorphous peaks used are listed in numerical order (Amorp.). Note that the WAXS experimental parameters are given in the main text whereas the crystallinity calculations are given in the supplementary information.

Time point	1 0 3	0 1 0	1 1 0 / 2 0 1	2 0 3	2 1 0	2 1 6	0 2 1	1 2 0	-2 1 2	-1 3 1	-2 2 4
**5 min**	-	-	16.41	-	-	-	-	18.39	-	-	-
**1 hr**	-	-	16.47	19.29	-	29.47	-	18.55	23.23	23.58	-
**2 hr**	-	-	16.79	19.25	22.46	29.76	12.71	-	23.47	-	-
**3 hr**	-	-	16.82	19.26	22.50	29.44	-	18.39	23.47	-	-
**6 hr**	-	14.78	16.82	19.24	22.52	29.26	-	18.39	23.45	-	-
**12 hr**	-	-	16.84	19.28	22.59	29.45	-	18.39	23.48	-	-
**24 hr**	12.52	14.98	16.76	19.15	20.89	29.28	-	-	23.08	23.42	-
**7 days**	12.38	-	16.81	19.21	22.52	29.32	-	18.39	23.07	23.45	27.10

Time point	Amorp. 1	Amorp. 2	Amorp. 3	Amorp. 4	Amorp. 5	Amorp. 6	% Area PEG Cryst.	% Area PLLA Cryst.	PLLA Cryst. (%)
**5 min**	17.90	21.64	30.00	-	-	-	1.9	19.4	31.4
**1 hr**	12.92	15.30	16.72	20.45	22.93	26.18	7.0	15.5	25.1
**2 hr**	10.93	15.30	18.39	20.65	23.01	26.18	7.1	26.9	43.5
**3 hr**	12.90	15.30	20.96	21.37	26.18	-	16.0	31.8	51.3
**6 hr**	12.20	15.30	20.47	21.93	23.07	27.18	13.6	40.6	65.7
**12 hr**	12.64	15.30	20.47	25.66	-	-	17.9	45.6	73.6
**24 hr**	12.73	19.39	21.93	26.52	-	-	23.8	50.3	81.4
**7 days**	14.25	15.14	20.44	23.08	-	-	18.7	52.0	84.0

Supplementary Table 7: Convoluted WAXS data with Voigt/Gaussian model for **15**: $PLLA_{90}$-*b*-PEG_{45} 10% solids w/w. Units are degrees unless otherwise stated. Peaks are color coded with the following: PLLA-green, PEG-yellow, amorphous-blue. Amorphous peaks used are listed in numerical order (Amorp.) Note that the WAXS experimental parameters are given in the main text whereas the crystallinity calculations are given in the supplementary information.

Time point	1 0 3	0 1 0	1 1 0 / 2 0 1	2 0 3	2 1 0	2 1 6	0 2 1	1 2 0	-2 1 2	-1 3 1	-2 2 4
**5 min**	-	-	16.36	-	-	-	-	18.39	-	-	-
**1 hr**	-	-	16.91	19.31	22.59	29.48	12.82	18.39	-	-	-
**3 hr**	-	-	16.90	19.27	22.58	29.36	-	18.39	23.51	-	-
**6 hr**	-	-	16.91	19.29	22.74	29.43	-	-	23.49	-	-
**24 hr**	-	-	16.93	19.31	22.56	29.41	-	18.39	22.93	-	-

Time point	Amorp. 1	Amorp. 2	Amorp. 3	Amorp. 4	Amorp. 5	Amorp. 6	Amorp. 7	% Area PEG Cryst.	% Area PLLA Cryst.	PLLA Cryst. (%)
**5 min**	14.01	20.91	22.50	-	-	-	-	2.0	18.9	24.7
**1 hr**	11.90	14.45	15.25	20.55	21.93	23.60	26.52	5.5	47.7	62.4
**3 hr**	12.03	14.31	15.25	20.57	23.13	27.18	-	3.4	52.3	68.4
**6 hr**	11.92	15.12	15.30	21.93	26.18	-	-	2.6	44.6	58.4
**24 hr**	10.69	12.71	14,25	15.19	23.74	27.18	-	5.6	63.2	82.7

SUPPLEMENTARY FIGURES

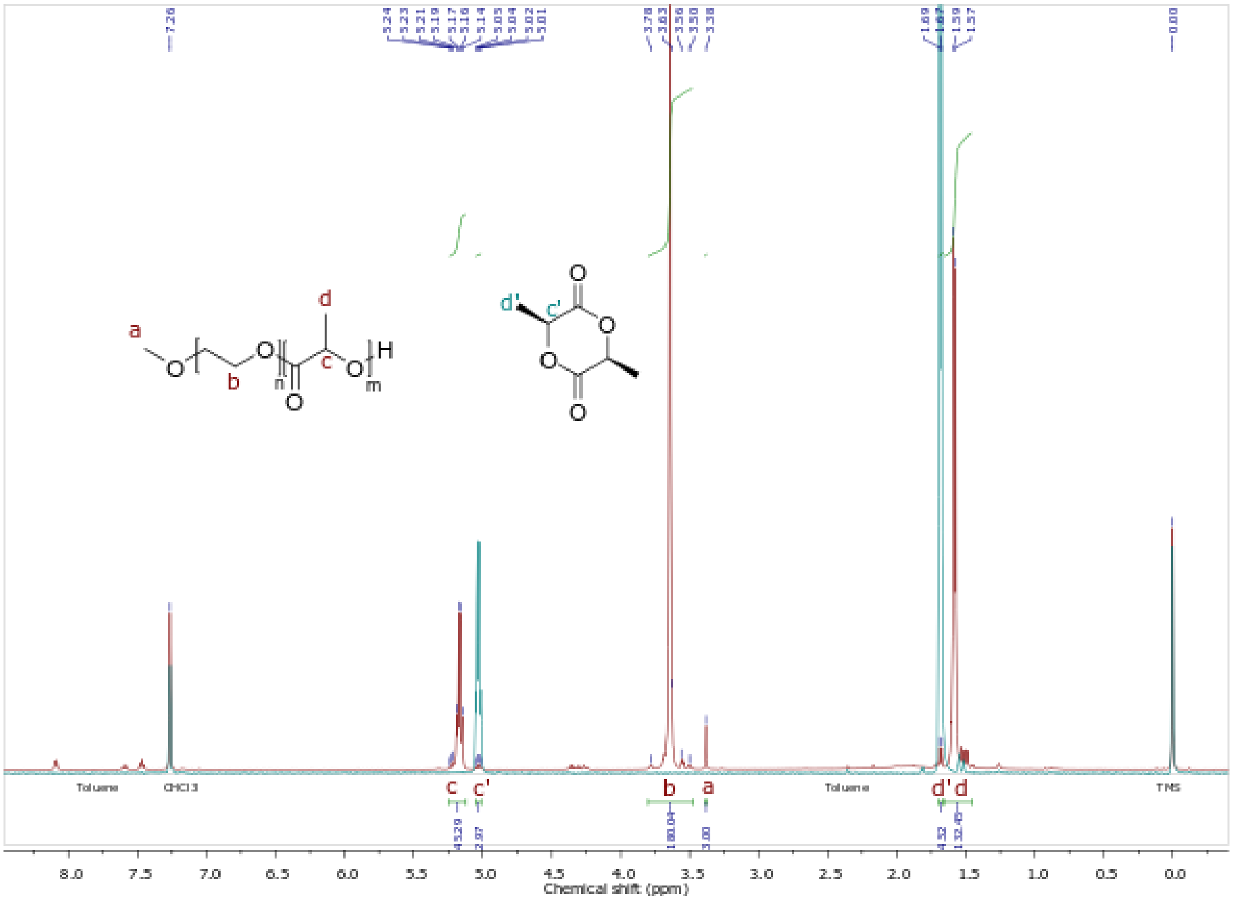

Supplementary Figure 1: Representative ^{1}H NMR spectrum for **7** (red,$PLLA_{45}$-*b*-PEG_{45} 5% solids w/w) overlaid with L-lactide (blue). Due to broad peak around 1.6, conversion was calculated from peaks around 5ppm. Conversion was then calculated from ^{1}H NMR data as shown in Supplementary Equation 3:

$$\%Conversion = \frac{Area\ of\ PLLA\ peak\ at\ 5.2}{Area\ of\ LL\ peak\ at\ 5.0 + Area\ of\ PLLA\ peak\ at\ 5.2} * 100\% \qquad (3)$$

For calculated ^{1}H NMR results see Supplementary Table 1 and for a list of peak assignments see the methods section.

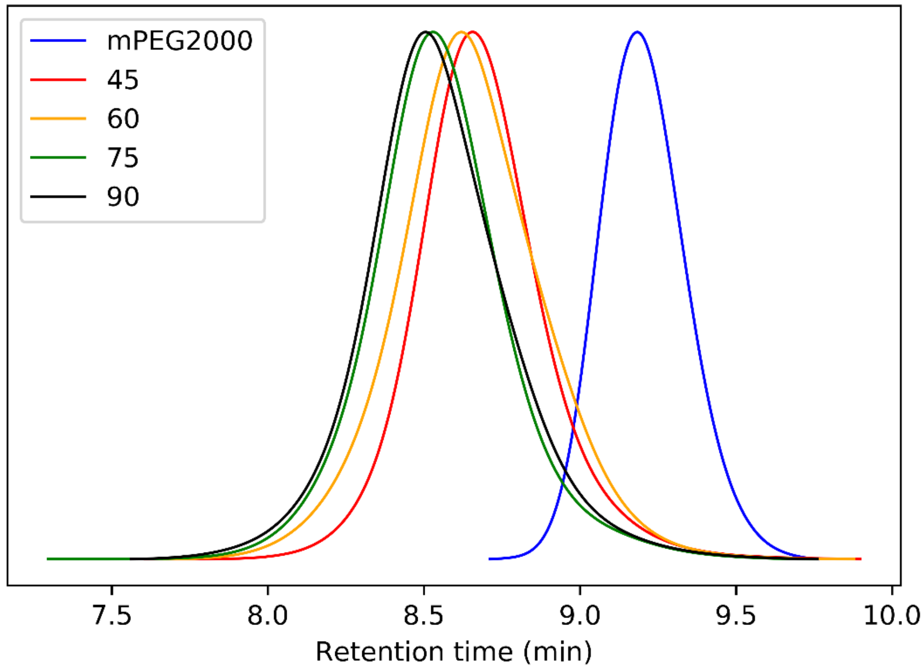

Supplementary Figure 2: Representative GPC traces for PLLA$_n$-*b*-PEG$_{45}$ for samples **9** (n=45), **11** (n=60), **12** (n=75), and **15** (n=90) all at 10% solids w/w. mPEG$_{45}$ (MW = 2000) is shown to prove chain extension from the macroinitiator. For the GPC results see Supplementary Table 1 and for the set-up see the methods section.

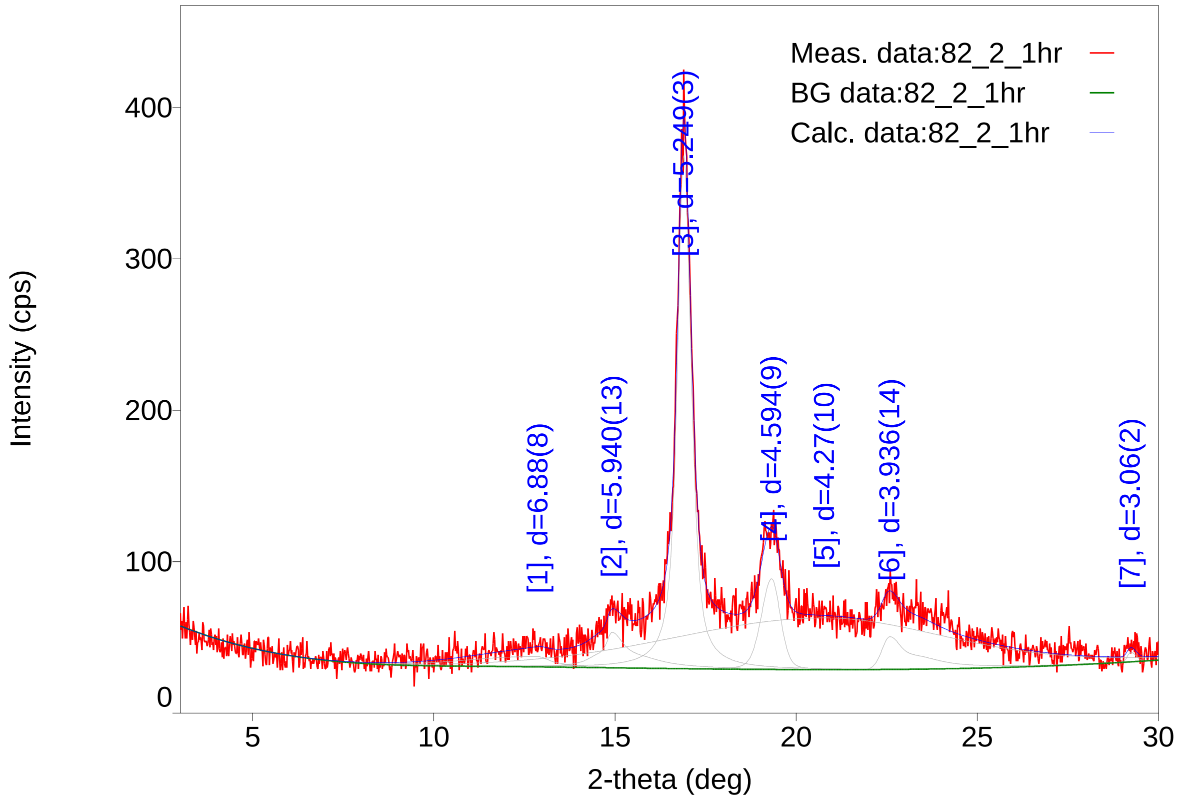

Supplementary Figure 3: Example of WAXS pattern peak selection for determination of % crystallinity. The green baseline represents the background. Peaks 1-4, 6-7 are crystalline peaks whereas peak 5 is the broad amorphous peak. The sample is **15** ($PLLA_{90}$-*b*-PEG_{45} 10% solids w/w) at 1-hour post polymerization. See Supplementary Tables 4 and 5 for peak assignments and crystallinity. See also methods section, results (section b) and Figure 2a-b in the main text for additional WAXS information and results.

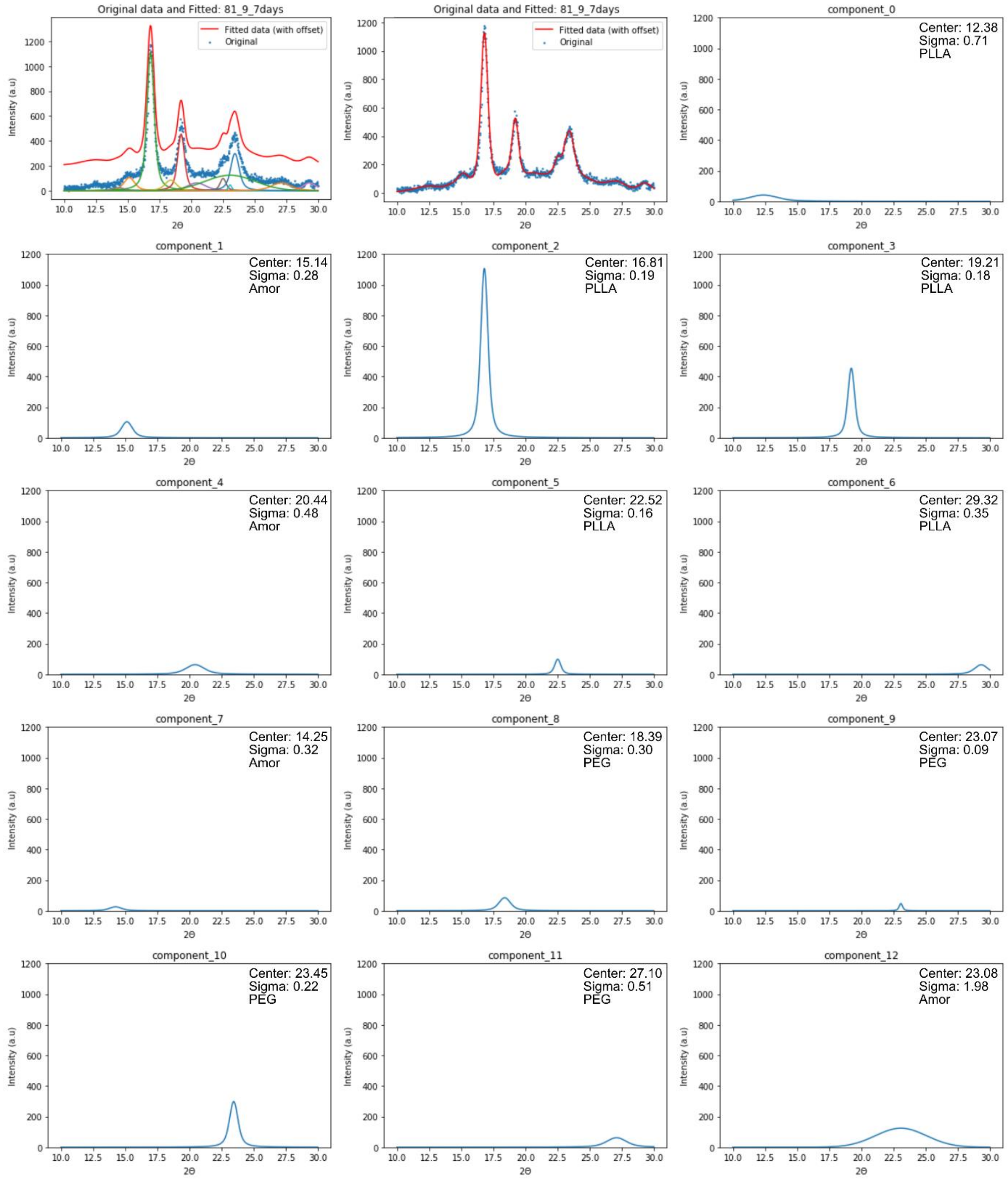

Supplementary Figure 4: Representative peak deconvolution which separates PLLA, PEG, and amorphous peaks enabling the tracking of PLLA and PEG crystallinity. The sample $PLLA_{45}$-*b*-PEG_{45} (10% solids w/w **9**) was taken seven days post polymerization. See Supplementary Tables 6 and 7 for peak assignments, integration, and crystallinity. See also methods section, results (section b) and Figure 2a-b in the main text for additional WAXS information and results.

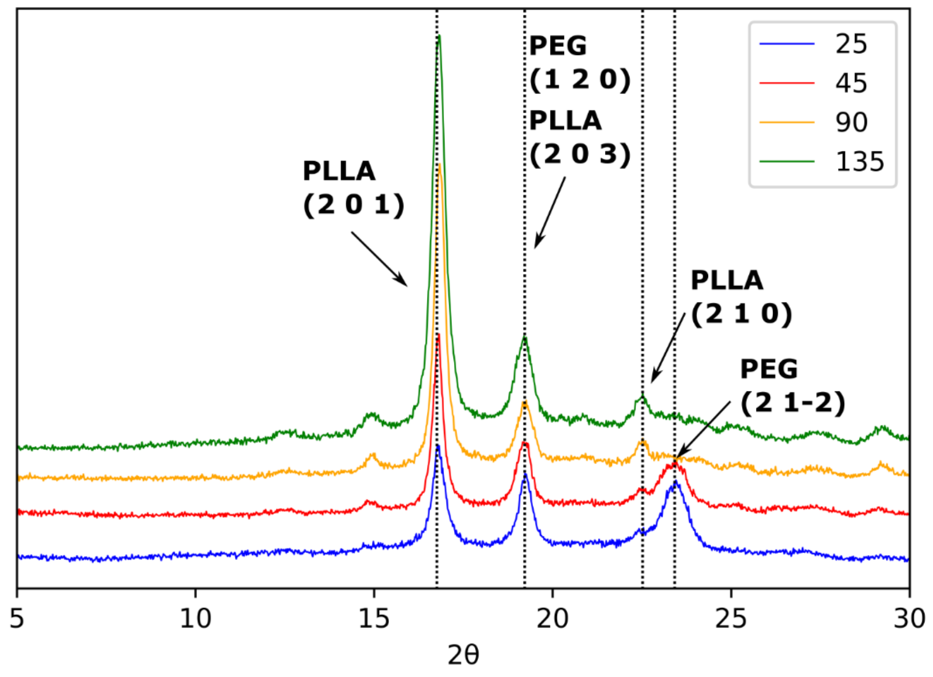

Supplementary Figure 5: WAXS diffraction patterns for $PLLA_{25}$-b-PEG_{45} (**6**), $PLLA_{45}$-b-PEG_{45} (**10**), $PLLA_{90}$-b-PEG_{45} (**16**), $PLLA_{135}$-b-PEG_{45} (**19**). All polymer samples were prepared from 20% solids w/w toluene solutions before freeze-drying. Dominant WAXS peaks for PLLA and PEG are labeled above showing that PEG peaks are much more prevalent at lower PLLA DPs. See also results (self-assembly and crystallization kinetics) in the main text.

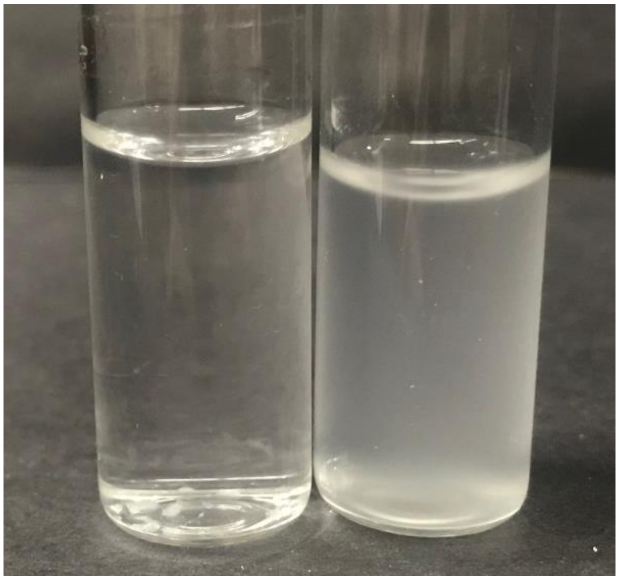

Supplementary Figure 6: Sample on the left is a control $PLLA_{90}$-*b*-PEG_{45} (synthesized in dichloromethane) that was sonicated in water for 30 mins. Notice the polymer precipitate at the bottom of the vial. Sample on the right is a ROPI-CDSA sample of $PLLA_{90}$-*b*-PEG_{45} that was lyophilized and then sonicated in water for 30 mins. Both samples have a PLLA DP=90 and the concentration of these samples is 0.5mg/mL or 0.5% solids w/w. This demonstrates that aqueous solutions are kinetically-trapped and that some microphase separation of PLLA and PEG (from self-assembly) is necessary to form stable nanoparticle based solutions.

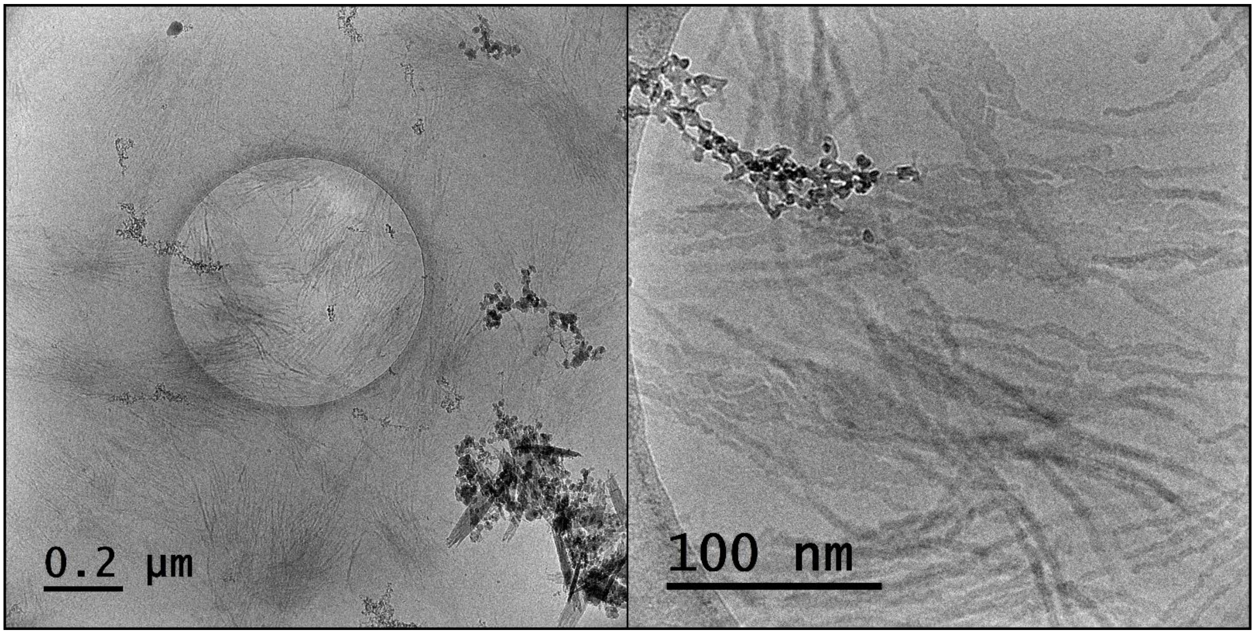

Supplementary Figure 7: Cryo-TEM images of $PLLA_{45}$-*b*-PEG_{45} worms resulting from solvent switch (5mg/mL) in water. The dark contrast in the bottom right of the left image and the top left of the right image corresponds to ice contamination. These structures are markedly different from structures obtained from ROPI-CDSA that have been transferred to water demonstrating that PLLA-*b*-PEG assemblies in pure water are likely kinetically-trapped.

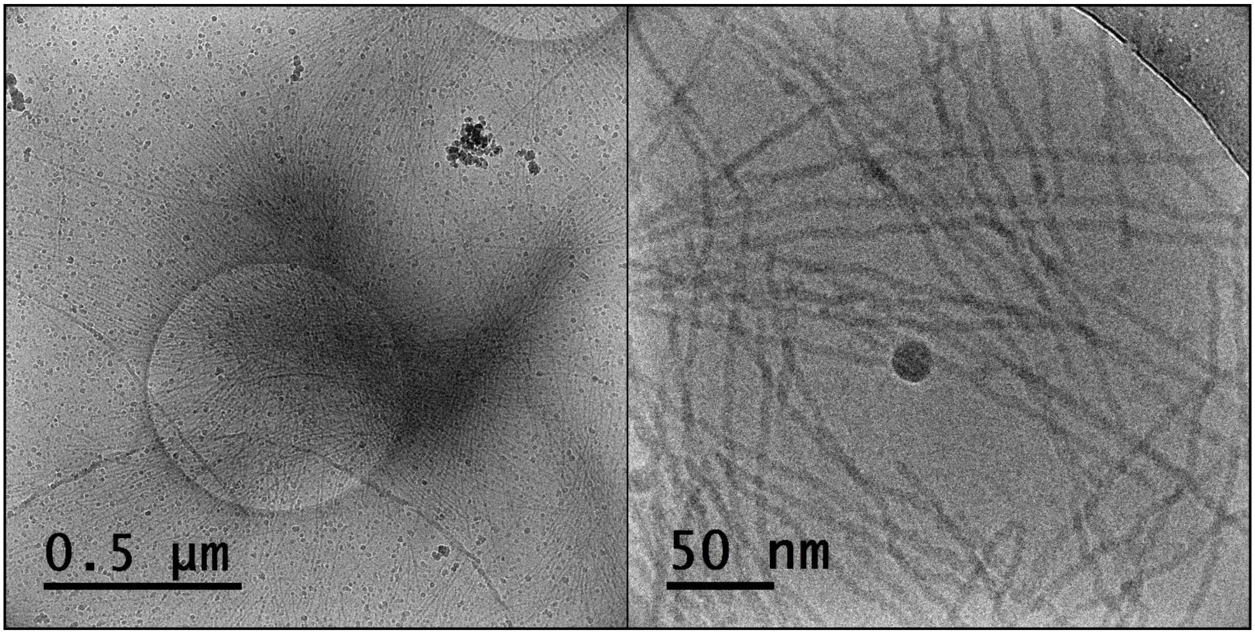

Supplementary Figure 8: Cryo-TEM images of $PLLA_{90}$-*b*-PEG_{45} worms/rods resulting from solvent switch (5mg/mL) in water. The dark contrast in the top right of the left image corresponds to ice contamination. These structures are markedly different from structures obtained from ROPI-CDSA that have been transferred to water demonstrating that PLLA-*b*-PEG assemblies in pure water are likely kinetically-trapped.

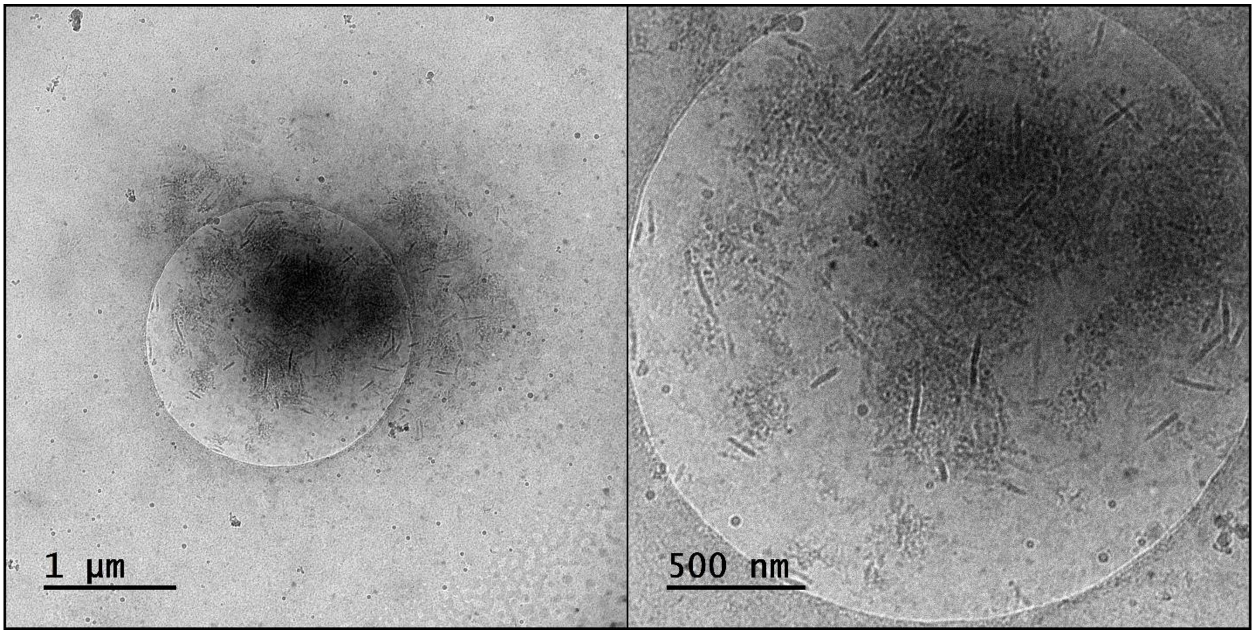

Supplementary Figure 9: Cryo-TEM images of $PLLA_{45}$-*b*-PEG_{45} particles resulting from CDSA in water showing short lamellar aggregations. These structures are markedly different from structures obtained from ROPI-CDSA that have been transferred to water. Additionally, CDSA only occurs in water upon heating of the sample to 65° C and letting it cool. This demonstrates that aqueous solutions of PLLA-*b*-PEG are kinetically trapped when left at room temperature.

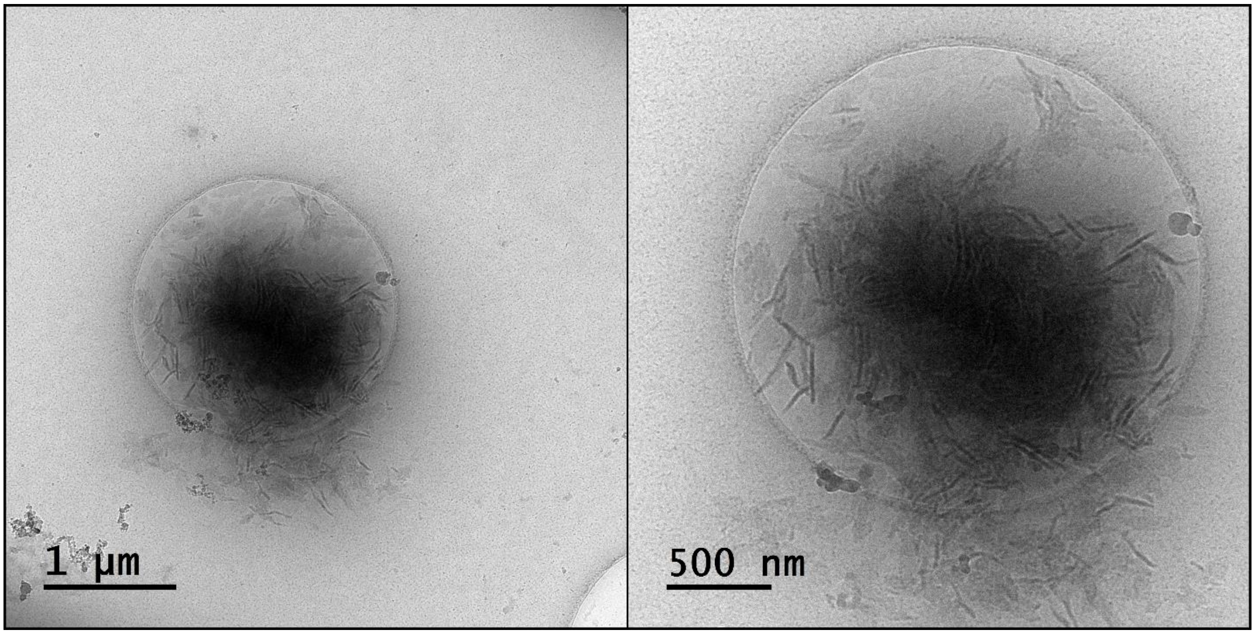

Supplementary Figure 10: Cryo-TEM images of $PLLA_{90}$-*b*-PEG_{45} particles resulting from CDSA in water showing short lamellae. These structures are markedly different from structures obtained from ROPI-CDSA that have been transferred to water. Additionally, CDSA only occurs in water upon heating of the sample to 65° C and letting it cool. This demonstrates that aqueous solutions of PLLA-*b*-PEG are kinetically trapped when left at room temperature.

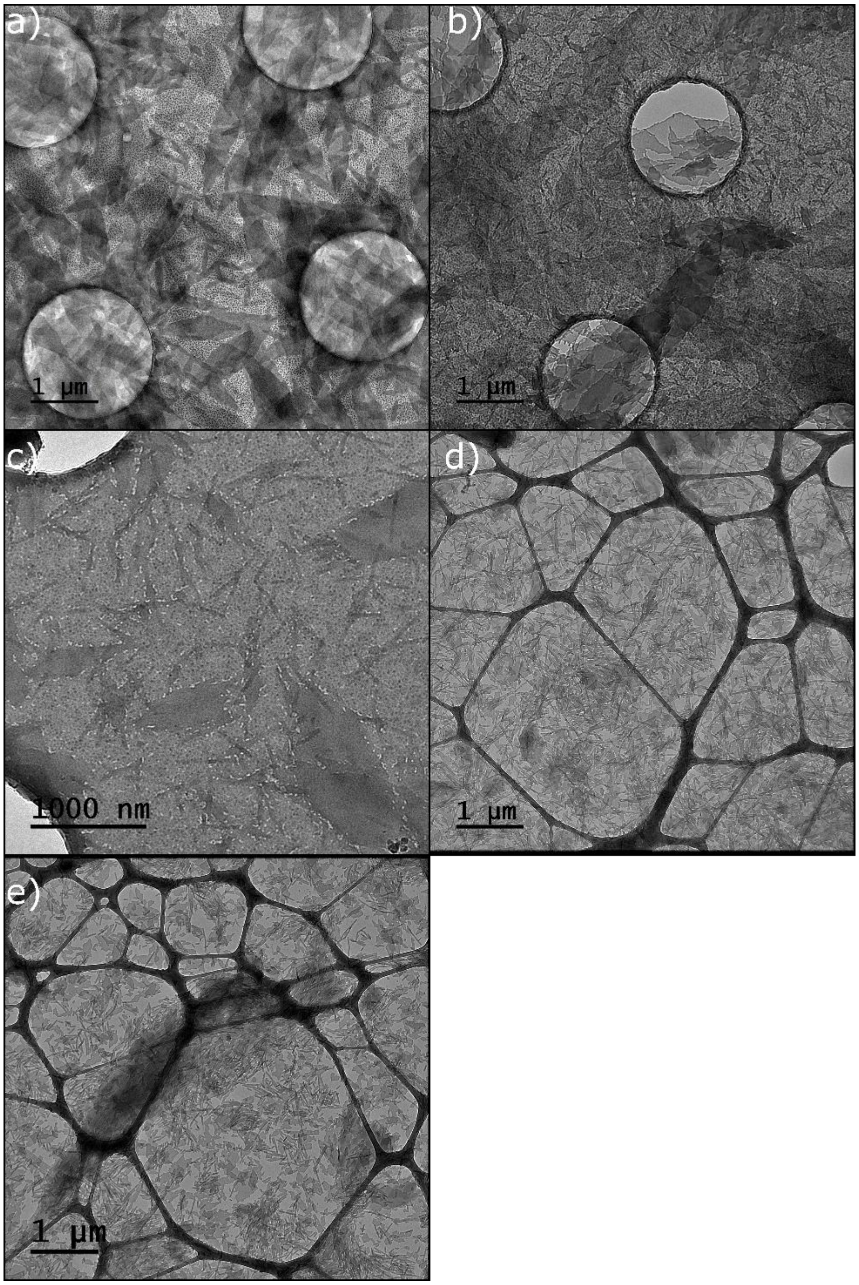

Supplementary Figure 11: Toluene cryo-TEM images. a) **9** (PLLA$_{45}$-*b*-PEG$_{45}$ 10% solids w/w) b) **13** (PLLA$_{90}$-*b*-PEG$_{45}$ 5% solids w/w) c) **9** d-e) graphene oxide coated grid with **9**. While images indicate the presence of lamellae and rods of various sizes, the ice layer is absent in (a-c) from the holes visible. Evaporation of toluene during grid preparation concentrated and dehydrated the sample. Dry-state TEM gives a similar result. Performing cryo-TEM on a graphene-oxide coated lacey carbon grid gave a better, albeit poor, ice layer. However, the solutions are still too concentrated to resolve structural features. Thus, toluene-based cryo-TEM prep would be replaced by water-based cryo-TEM. For information on water-based cryo-TEM sample preparation, see the methods section.

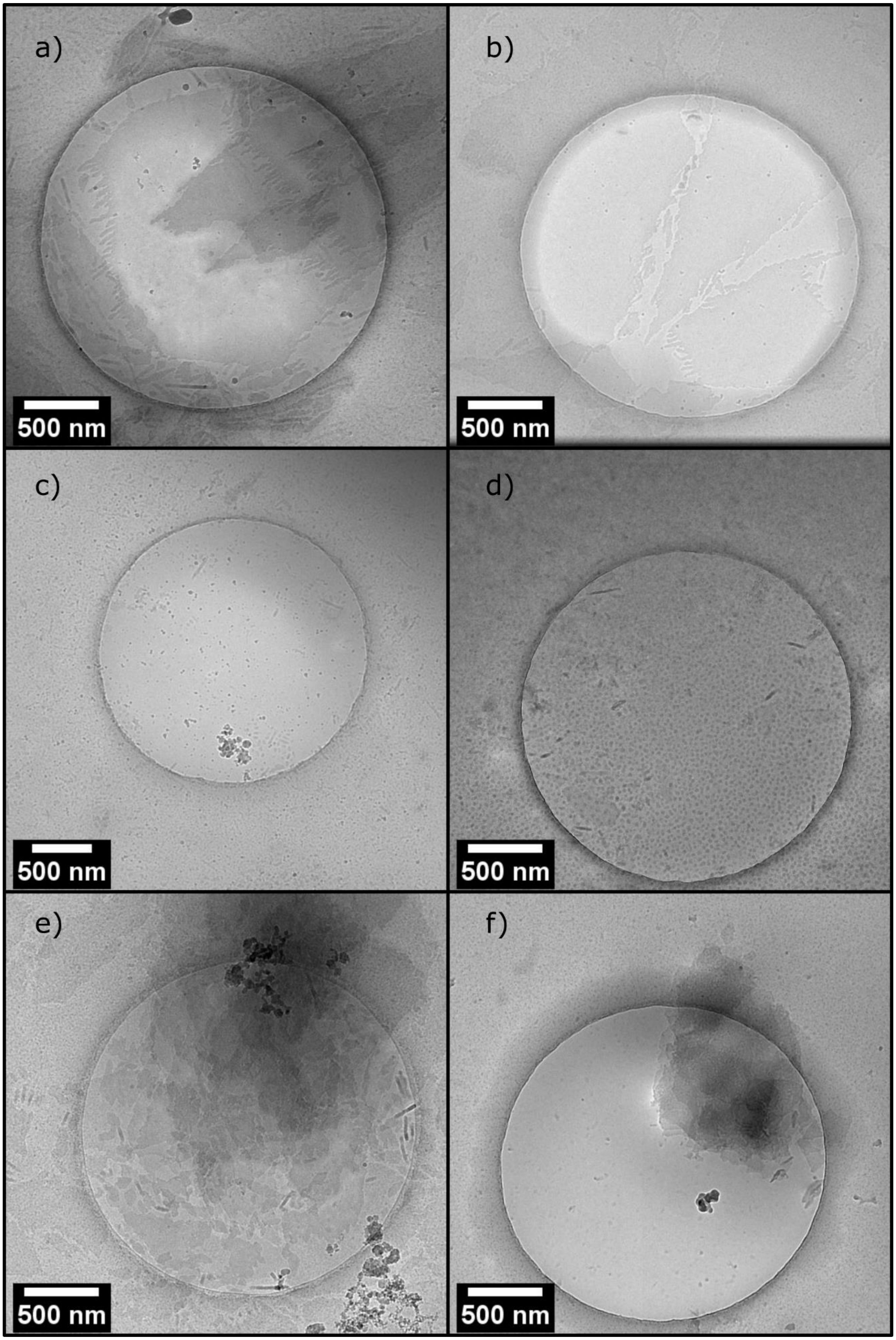

a)
500 nm
b)
500 nm
c)
500 nm
d)
500 nm
e)
500 nm
f)
500 nm

Supplementary Figure 12: Cryo-TEM images of aqueous resuspensions that were taken shortly after resuspensions (a,c,e), months after resuspension (b), and from resuspensions made from aged freeze-dried samples over six months aged (d, f). Images (a) and (b) are lamellae from $PLLA_{75}$-*b*-PEG_{45} (10% solids w/w **12**) resuspensions relaxed several days in toluene prior to resuspension. Images (c) and (d) are nanorods and spheres from $PLLA_{45}$-*b*-PEG_{45} (10% solids w.w **9**) resuspensions relaxed six hours in toluene prior to freeze-drying. Images (e) and (f) are lamellae aggregates from $PLLA_{90}$-*b*-PEG_{45} relaxed one day in toluene prior to freeze-drying. In each case, the morphologies are maintained showing that PLLA-*b*-PEG morphologies are stable and kinetically-trapped once freeze-dried and transferred to water. For more information see section **c**, self-assembly mechanism, in the results.

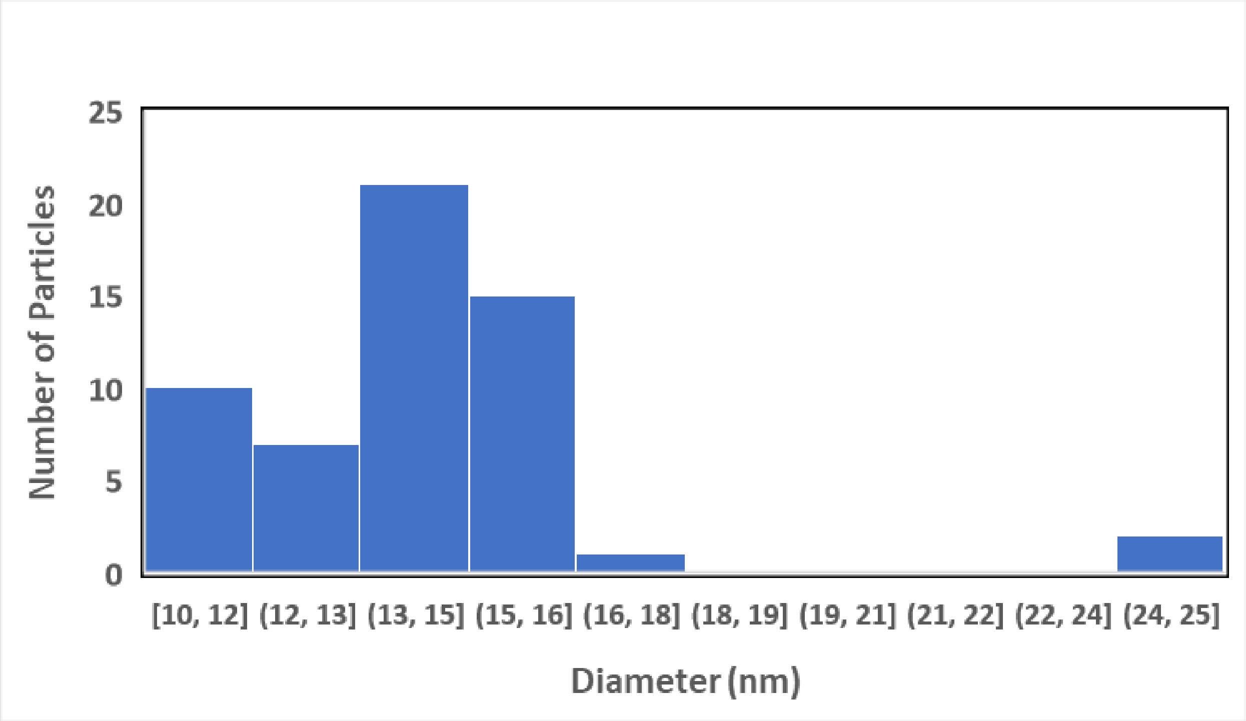

Supplementary Figure 13: Histogram for the sphere diameter (14.4±2.6 nm) of $PLLA_{45}$-b-PEG_{45} measured t = 1 hour of self-assembly from cryo-TEM. See also the results section (self-assembly mechanism) in the main text.

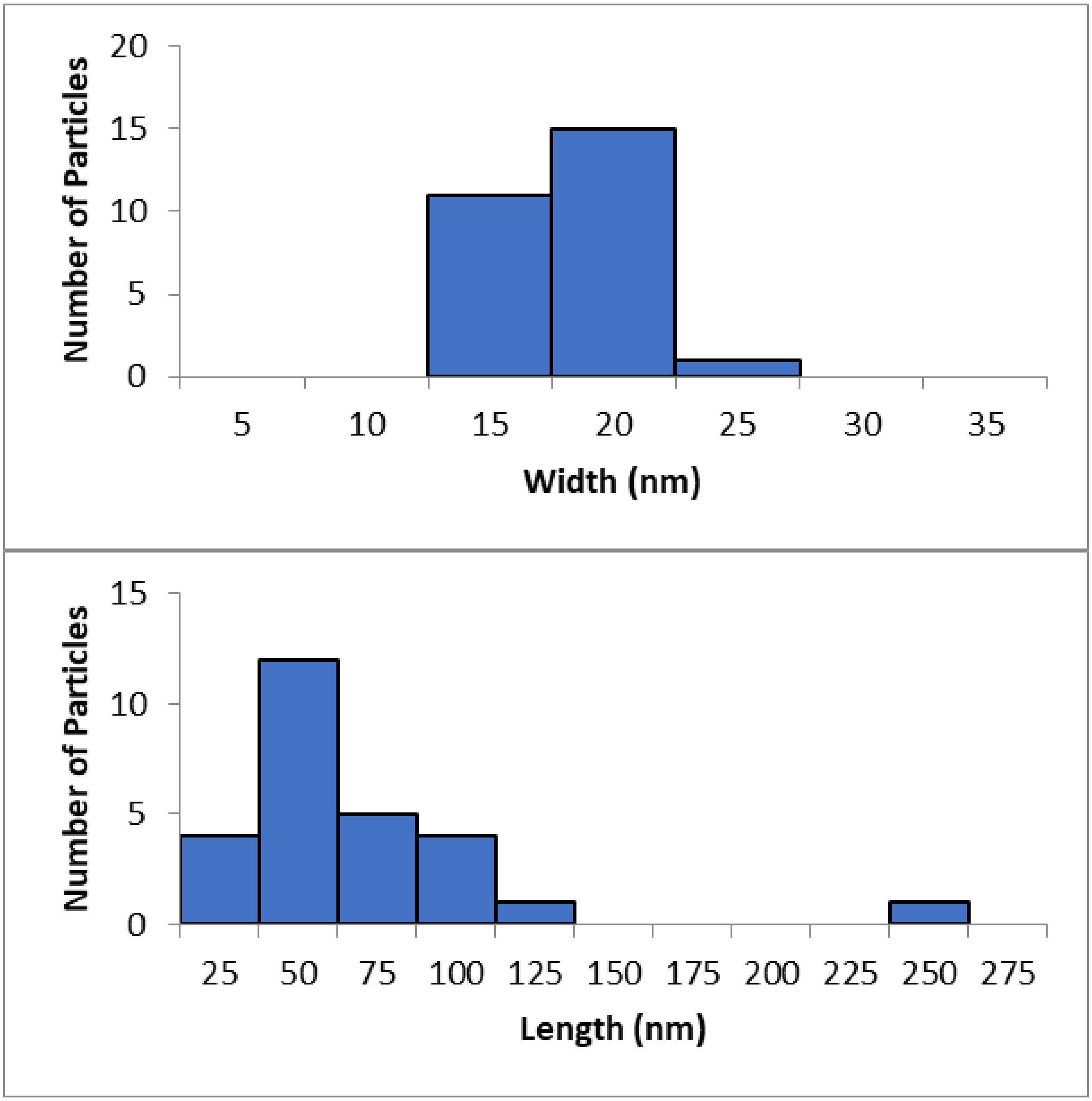

Supplementary Figure 14: Histograms for the rod width/diameter (15.8±2.3) (top) and length (56.1±42.4 nm) (bottom) of $PLLA_{45}$-b-PEG_{45} measured t = 3 hours of self-assembly from cryo-TEM. See also the results section (self-assembly mechanism) in the main text.

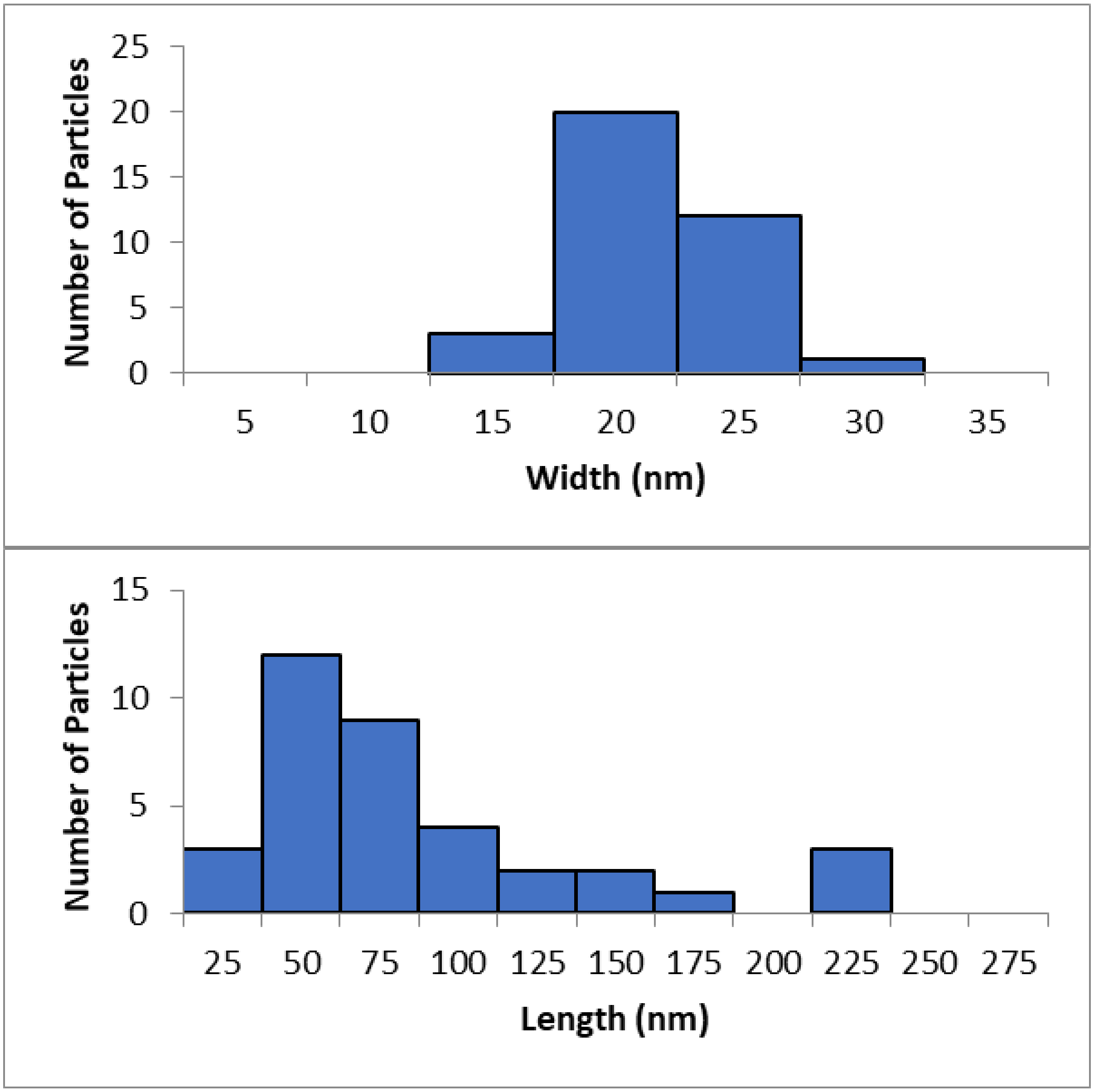

Supplementary Figure 15: Histograms for the rod width/diameter (19.1±3.1 nm) (top) and length (75.2±53.6 nm) (bottom) of $PLLA_{45}$-b-PEG_{45} measured t = 6 hours of self-assembly from cryo-TEM. See also the results section (self-assembly mechanism) in the main text.

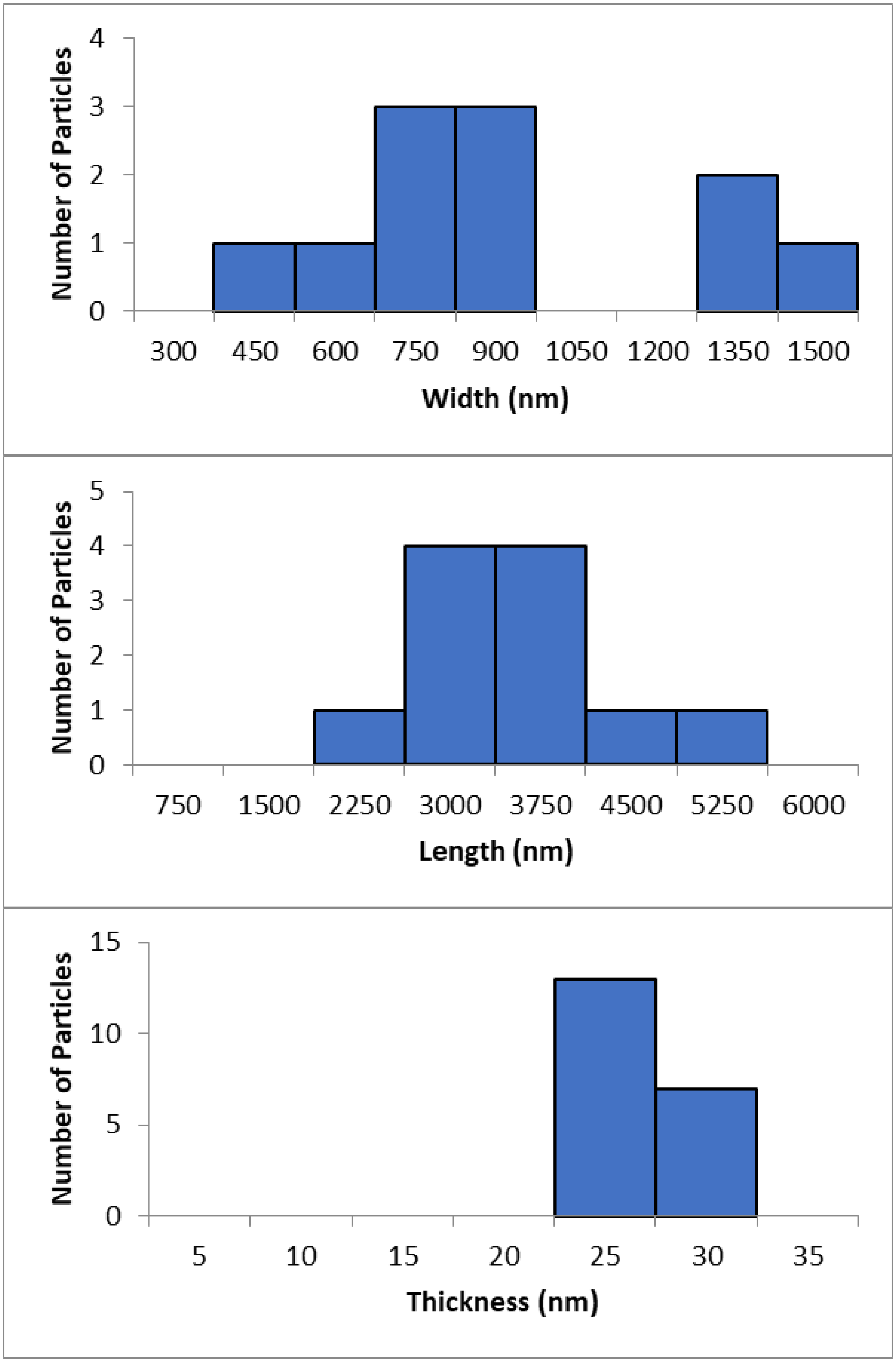
Number of Particles
Width (nm)
300 450 600 750 900 1050 1200 1350 1500
Number of Particles
Length (nm)
750 1500 2250 3000 3750 4500 5250 6000
Number of Particles
Thickness (nm)
5 10 15 20 25 30 35

Supplementary Figure 16: Histograms for the lamellae width (859±308 nm) (top), length (3212±927 nm) (middle) and thickness (24.7±2.2 nm) (bottom) of $PLLA_{45}$-b-PEG_{45} measured t = 24 hours of self-assembly from cryo-TEM. See also the results section (self-assembly mechanism) in the main text.

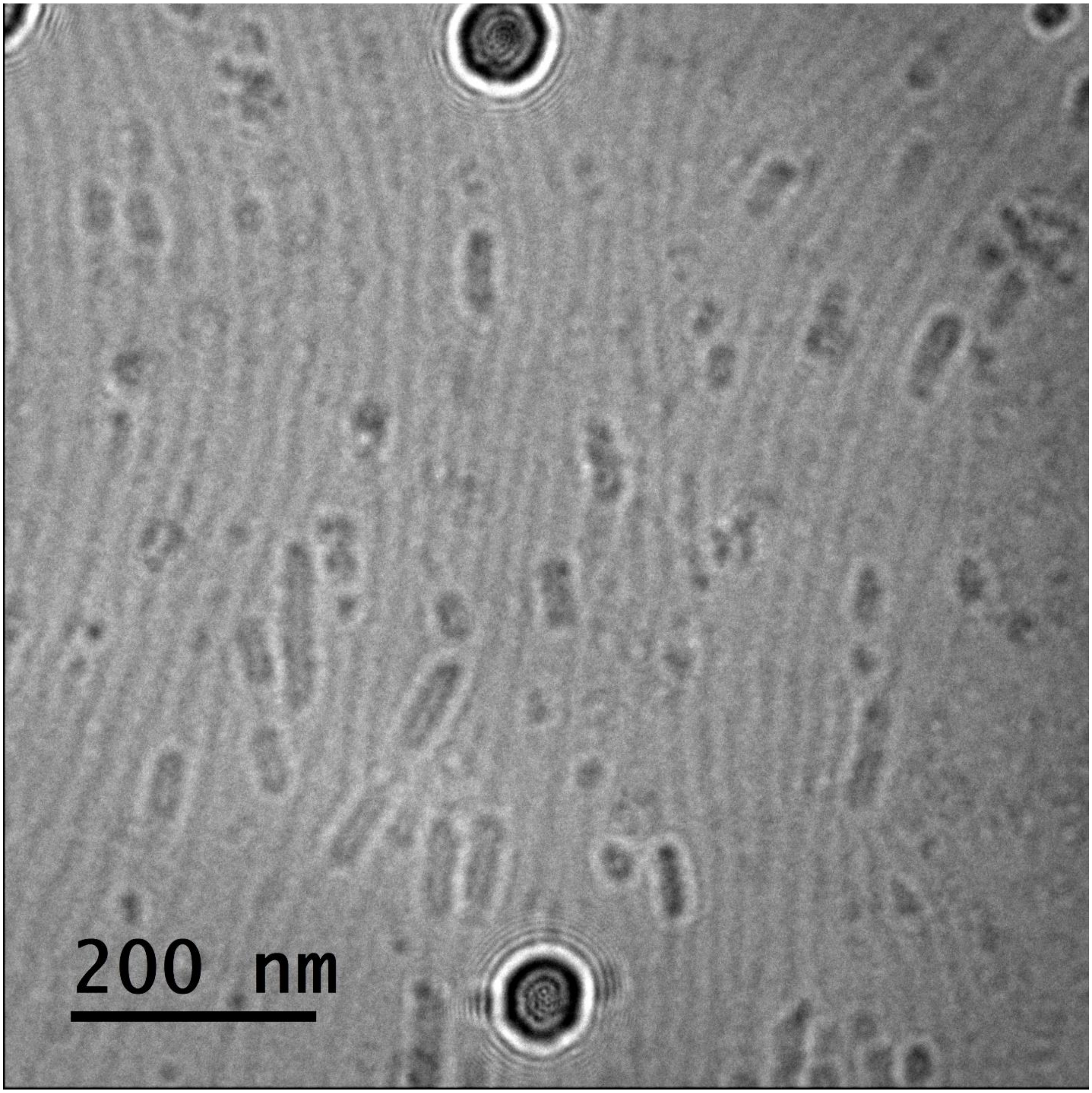

Supplementary Figure 17: Cryo-TEM image of fibers observed in a sample of $PLLA_{45}$-*b*-PEG_{45} (10% solids w/w **9**) that is primarily rods. Rods appear to be growing from fibers of a diameter of approximately 10 nm. The spheres which are highly defocused are ice contamination. Here fibers are defined as long thin nanostructures whereas rods are short and more defined.

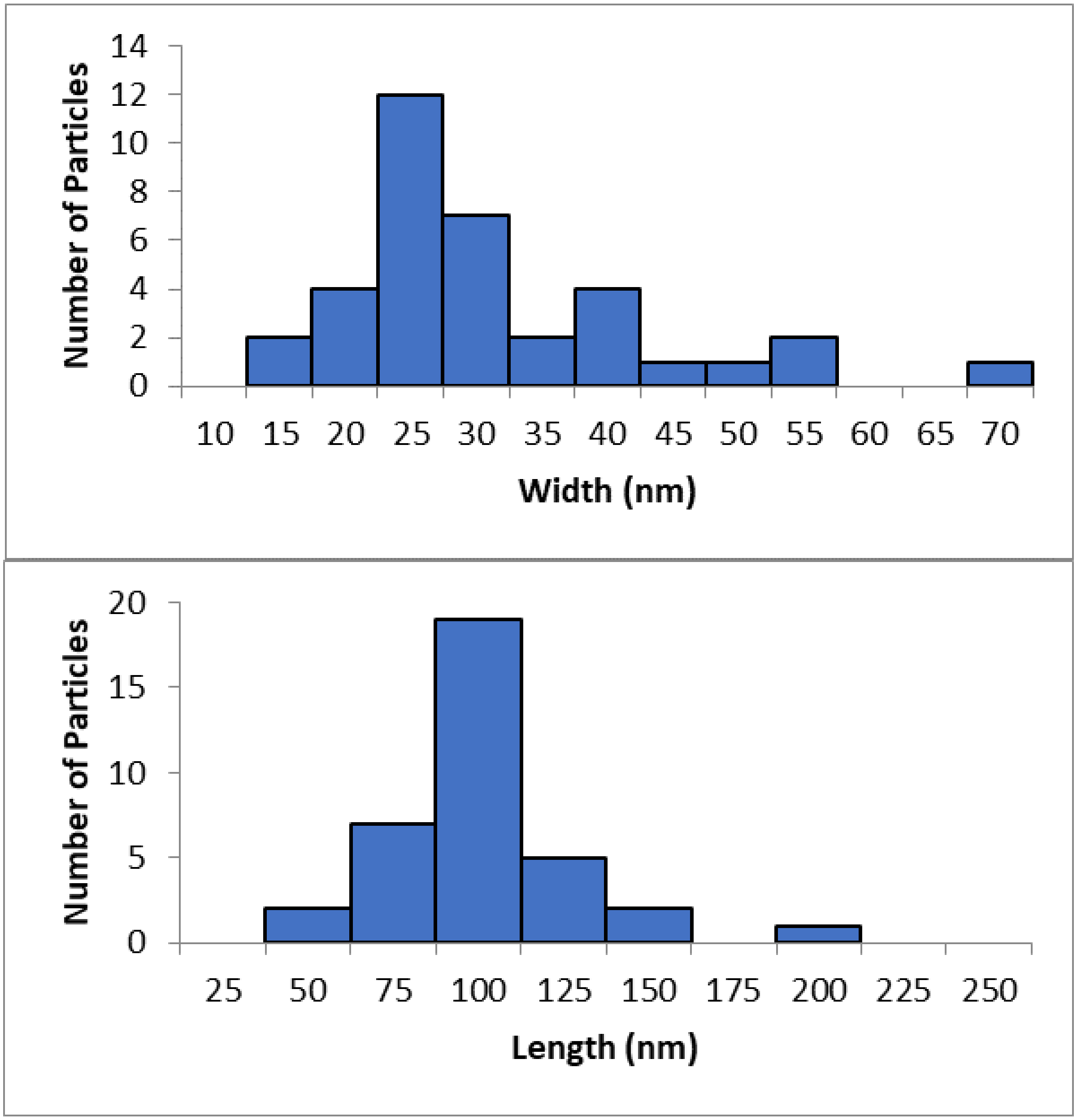

Supplementary Figure 18: Histograms for the rod-like precursor width/diameter (29±12 nm) (top) and length (90±27 nm) (bottom) of $PLLA_{90}$-b-PEG_{45} measured t = 5 min of self-assembly from cryo-TEM. See also the results section (self-assembly mechanism) in the main text.

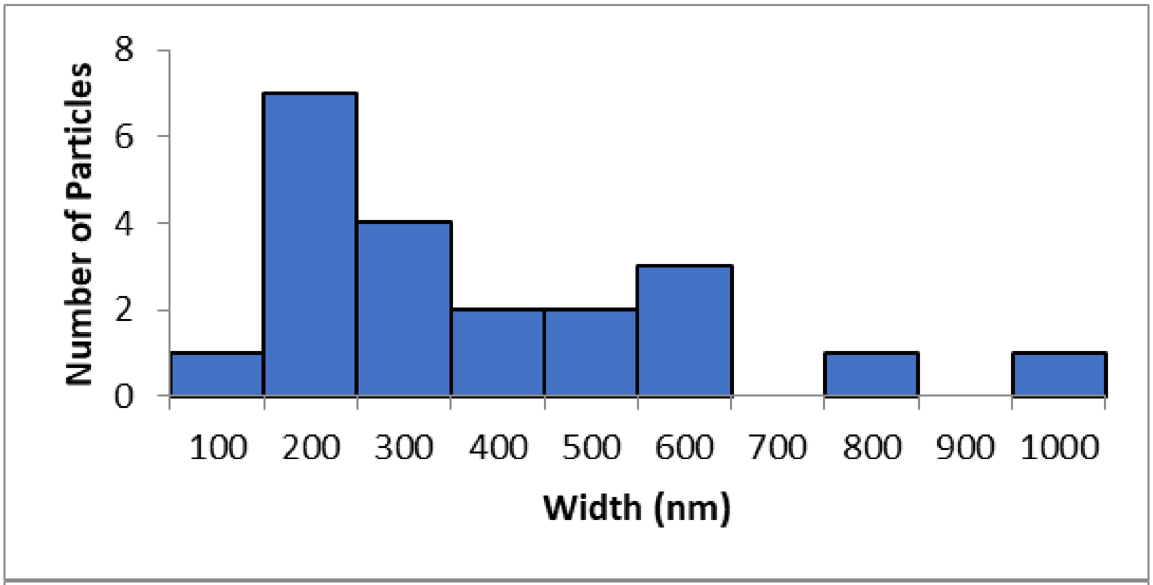
Number of Particles
8
6
4
2
0
100 200 300 400 500 600 700 800 900 1000
Width (nm)

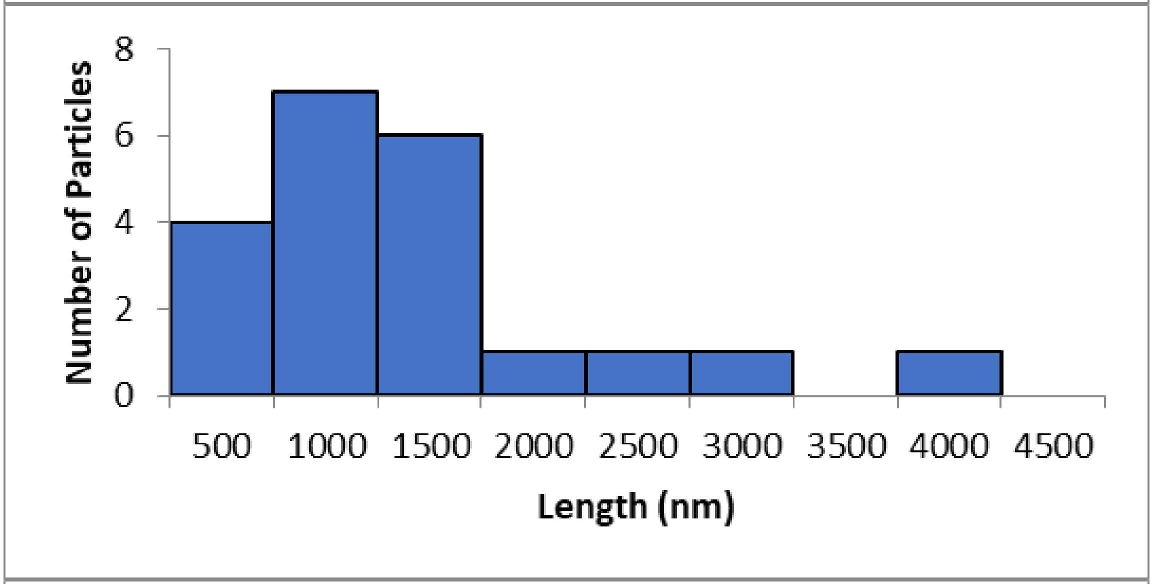
Number of Particles
8
6
4
2
0
500 1000 1500 2000 2500 3000 3500 4000 4500
Length (nm)

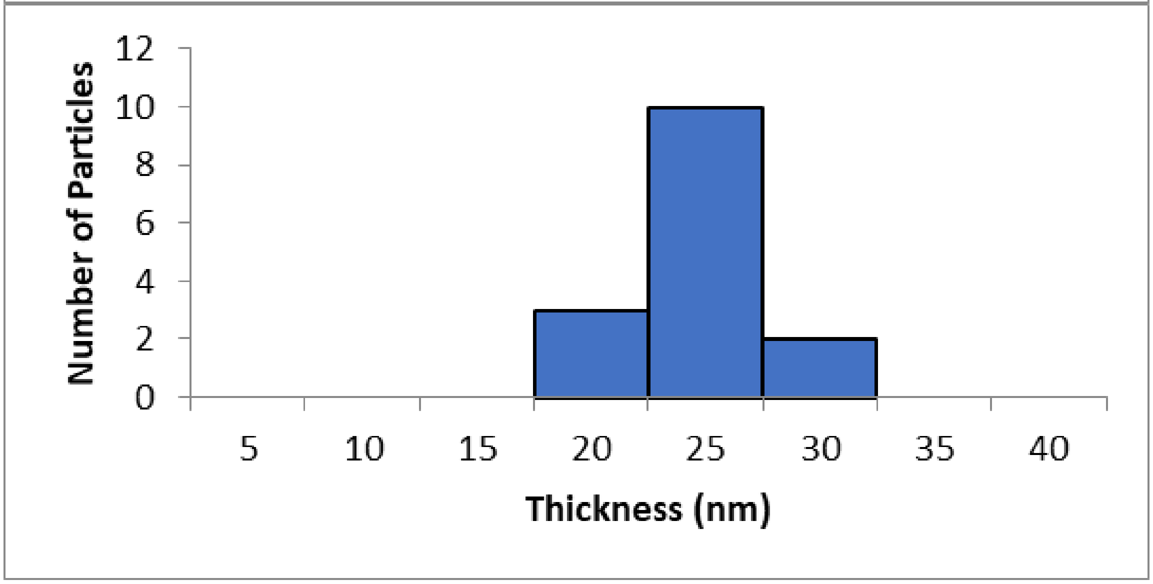
Number of Particles
12
10
8
6
4
2
0
5 10 15 20 25 30 35 40
Thickness (nm)

Supplementary Figure 19: Histograms for the lamellae width (360±250 nm) (top), length (1270±910 nm) (middle) and thickness (22±2 nm) (bottom) of $PLLA_{90}$-b-PEG_{45} measured t = 3 hours of self-assembly from cryo-TEM. See also the results section (self-assembly mechanism) in the main text.

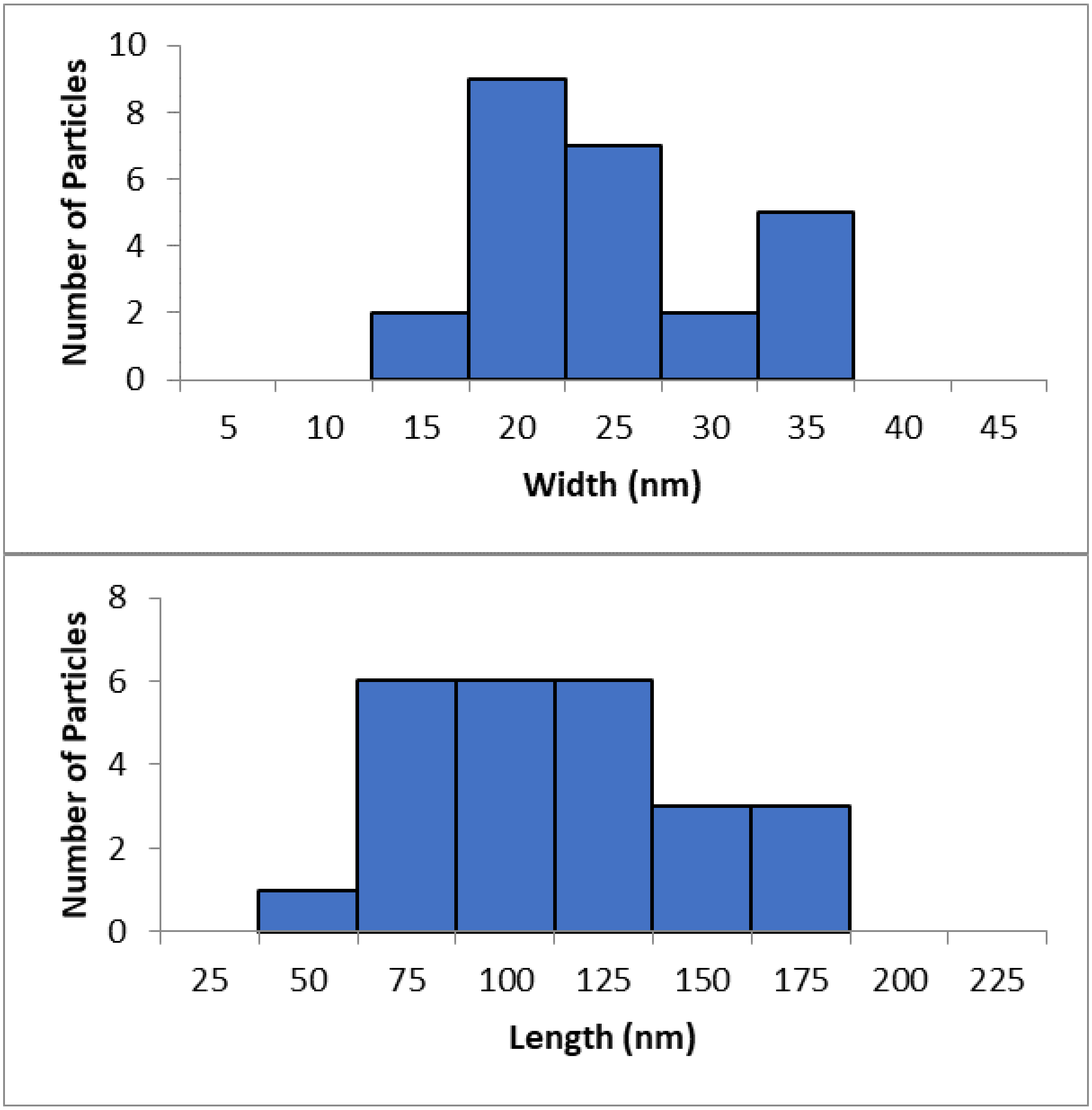

Supplementary Figure 20: Histograms for the rod width/diameter (22±6 nm) (top) and length (101±35 nm) (bottom) of $PLLA_{90}$-b-PEG_{45} measured t = 3 hours of self-assembly from cryo-TEM. Also see the results section (self-assembly mechanism) in the main text.

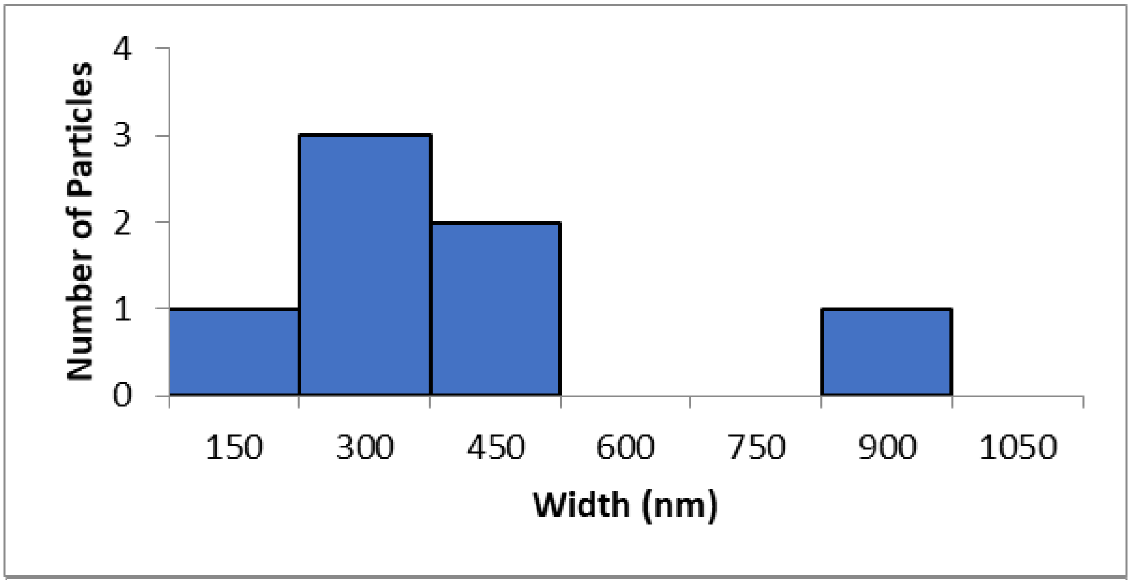

Number of Particles
4
3
2
1
0
150
300
450
600
750
900
1050
Width (nm)

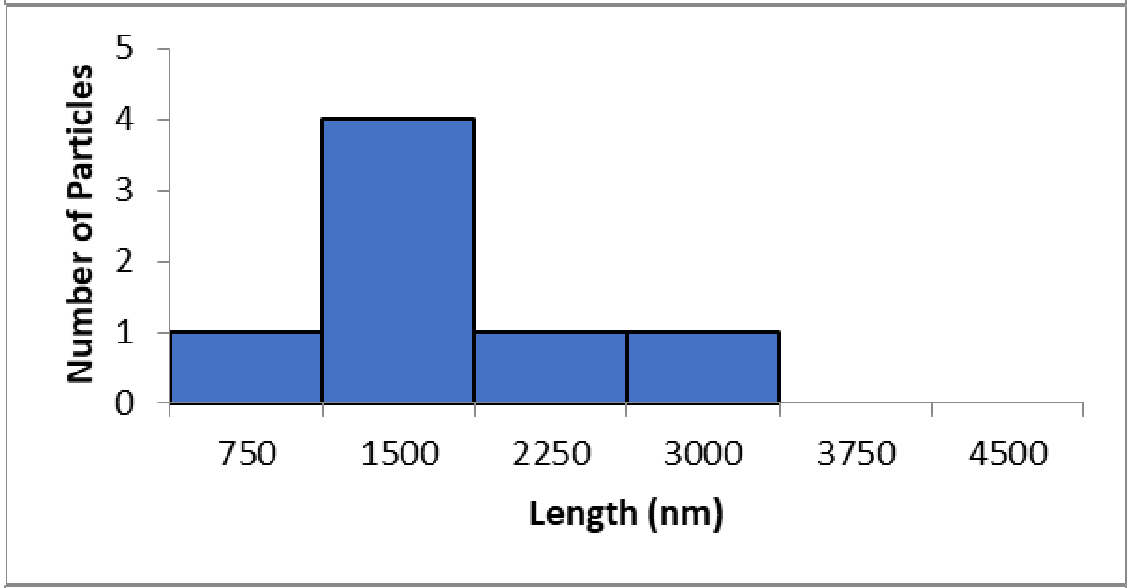

Number of Particles
5
4
3
2
1
0
750
1500
2250
3000
3750
4500
Length (nm)

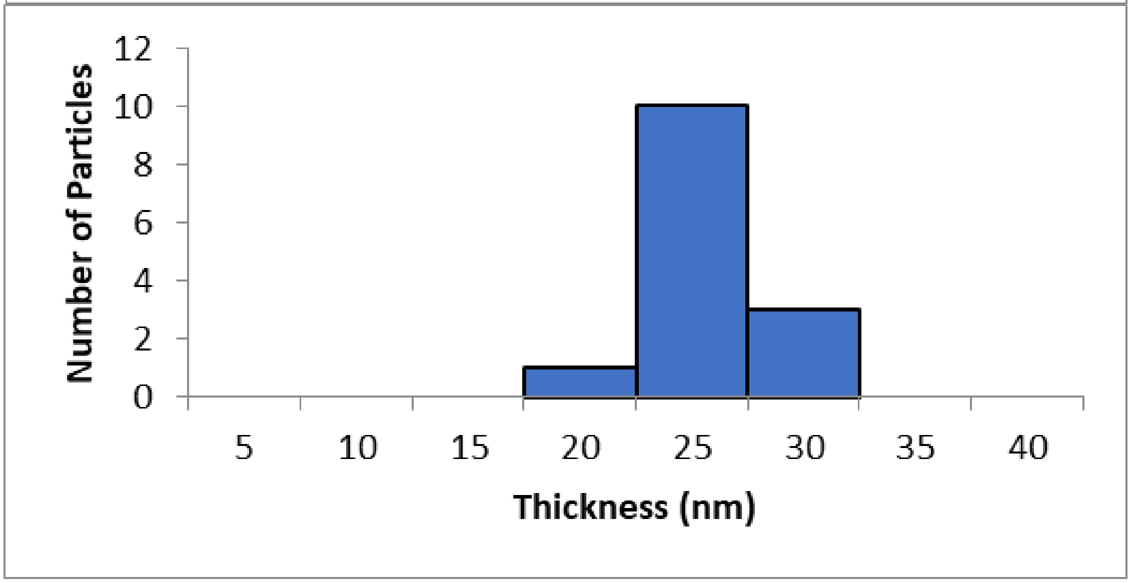

Number of Particles
12
10
8
6
4
2
0
5
10
15
20
25
30
35
40
Thickness (nm)

Supplementary Figure 21: Histograms for the lamellae width (360±230 nm) (top), length (1300±700 nm) (middle) and thickness (23±2 nm) (bottom) of $PLLA_{90}$-b-PEG_{45} measured t = 6 hours of self-assembly from cryo-TEM. Also see the results section (self-assembly mechanism) in the main text.

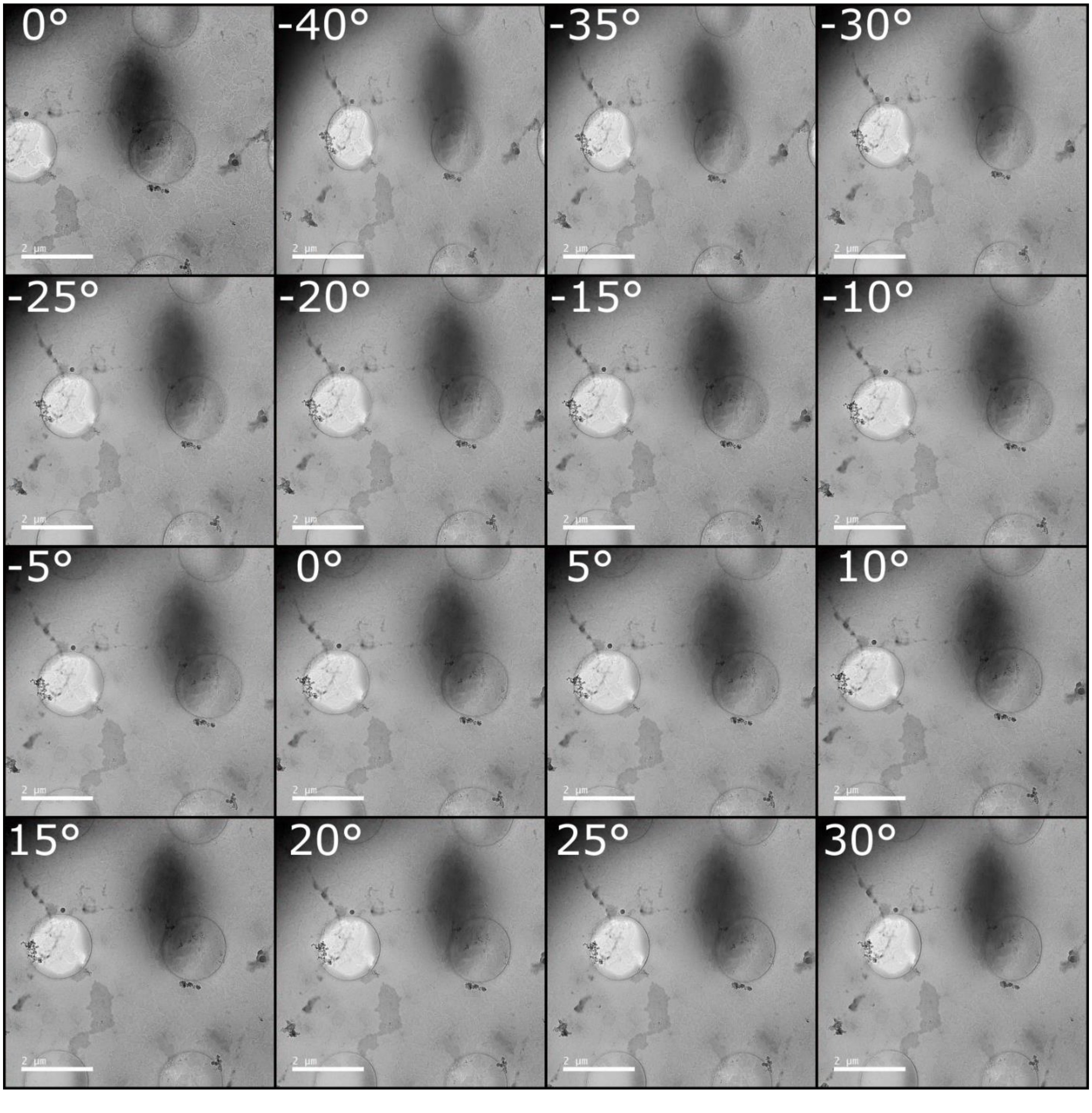
0°
-40°
-35°
-30°
-25°
-20°
-15°
-10°
-5°
0°
5°
10°
15°
20°
25°
30°
2 µm

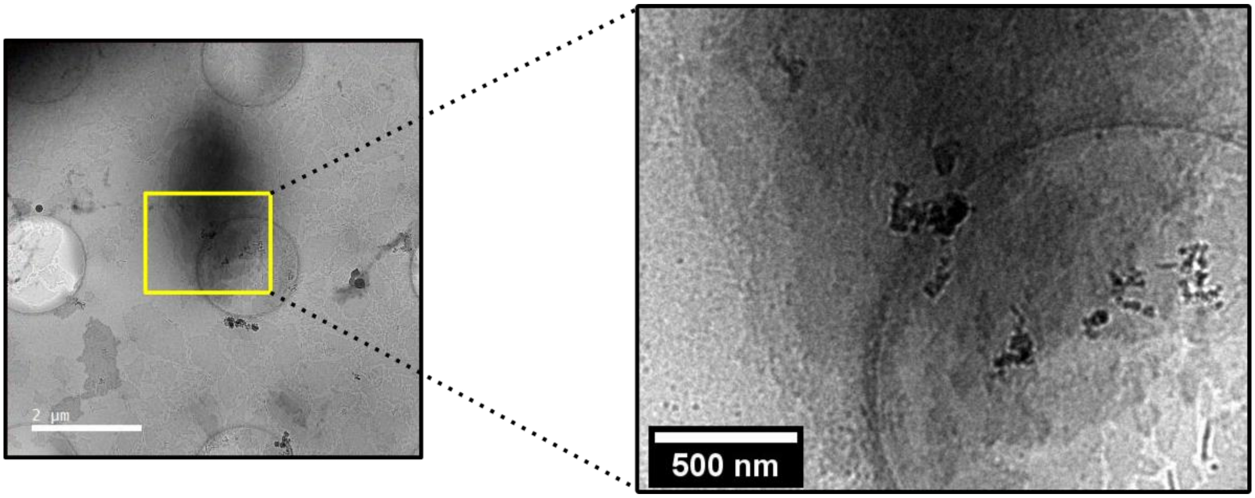

Supplementary Figure 22: (top) Cryo-TEM images of a tilt Angle series of a lamellar stack **15** ($PLLA_{90}$-*b*-PEG_{45} 10% solids w/w). Scale bar is 2 µm. (bottom) Zoomed in cryo-TEM image of lamellae in tilt angle series at 0°. For more information on lamellae stacking see section c, self-assembly mechanism and section d, phase diagram, in the results section.

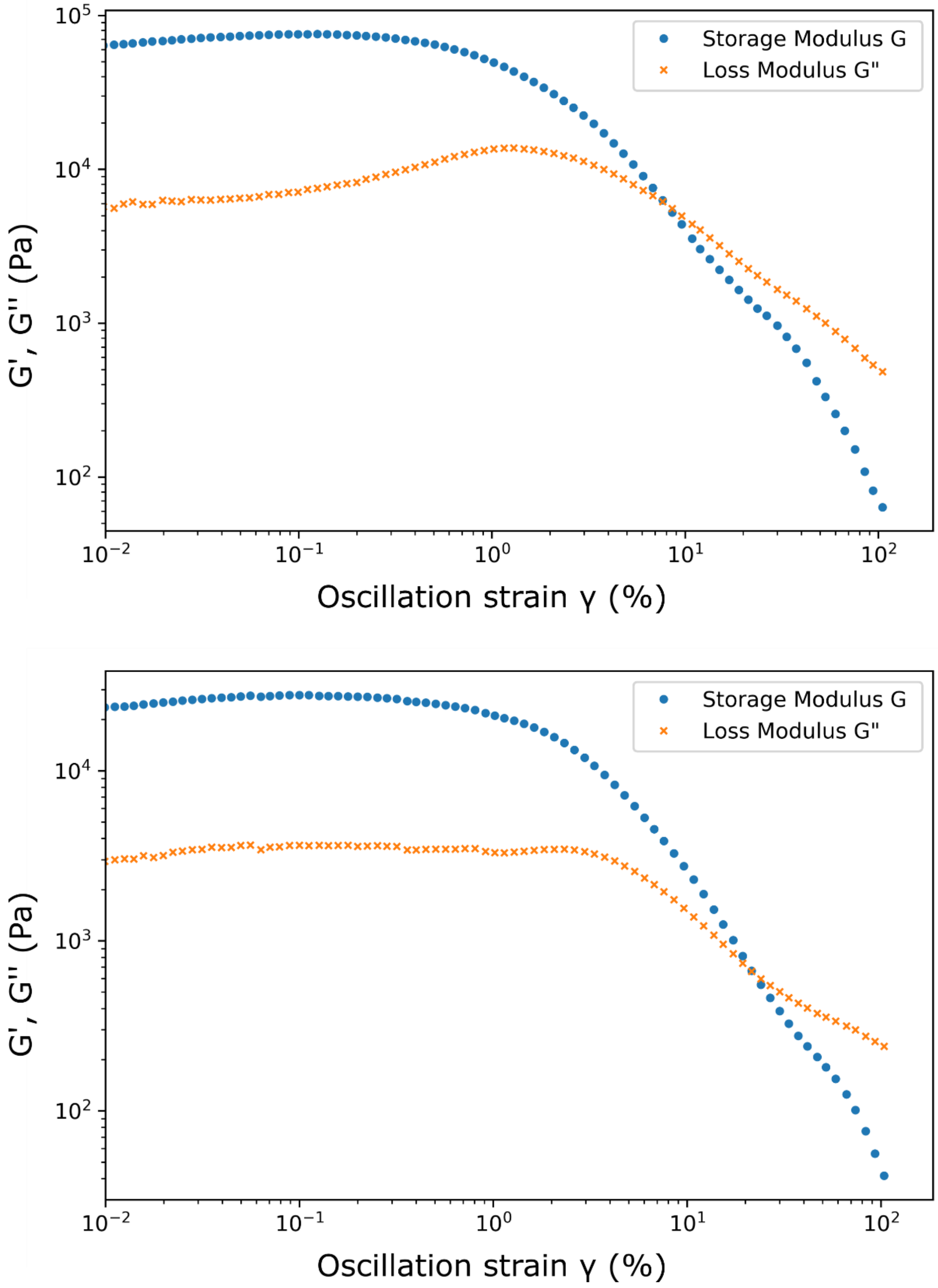

Supplementary Figure 23: Oscillatory rheology for organogels from triplicate runs of PLLA$_{90}$-*b*-PEG$_{45}$ (20% solids w/w **16**, top) and <u>PLLA$_{135}$-*b*-PEG$_{45}$</u> (20% solids w/w **19**, bottom). The crossover of the loss modulus and the storage modulus indicate the point of gelation. The point of gelation of **16** and **19** are about 8 and 10 γ(%) respectively. Polymers at higher PLLA DP (90 and above) and/or higher % solids w/w (10-20) form into organogels including **15**-**19**.

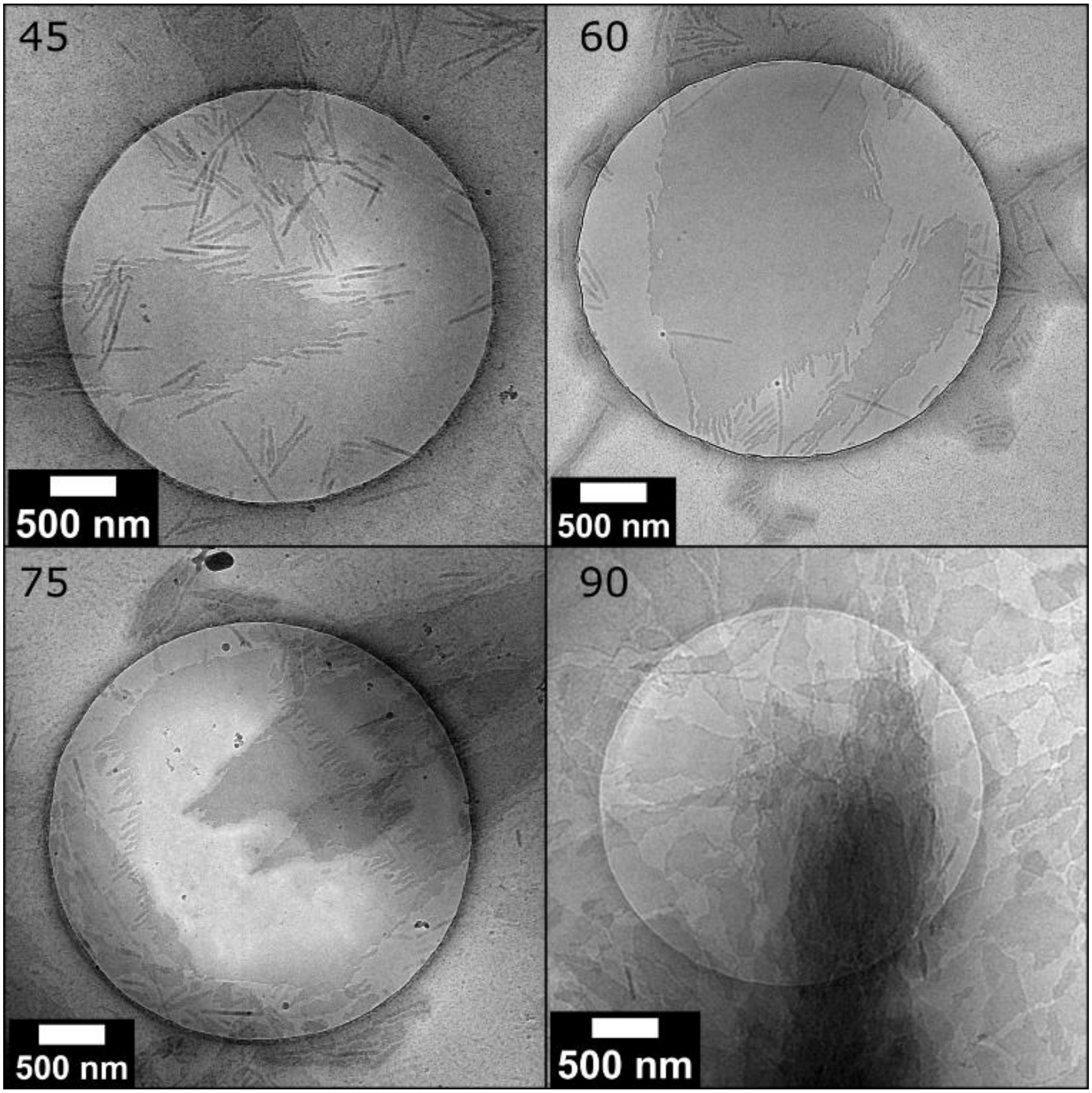

Supplementary Figure 24: Cryo-TEM images of relaxed PLLA$_n$-*b*-PEG$_{45}$ samples: **9** (n=45), **11** (n=60), **12** (n=75), and **15** (n=90) all at 10% solids w/w**11** and **12** lamellae exhibit similarities to **11** in that they form from rods, but they appear less ordered such as the lamellae in **15**. For further information see the turbidity data in Figure 1 and the discussion (section a) in the main text.

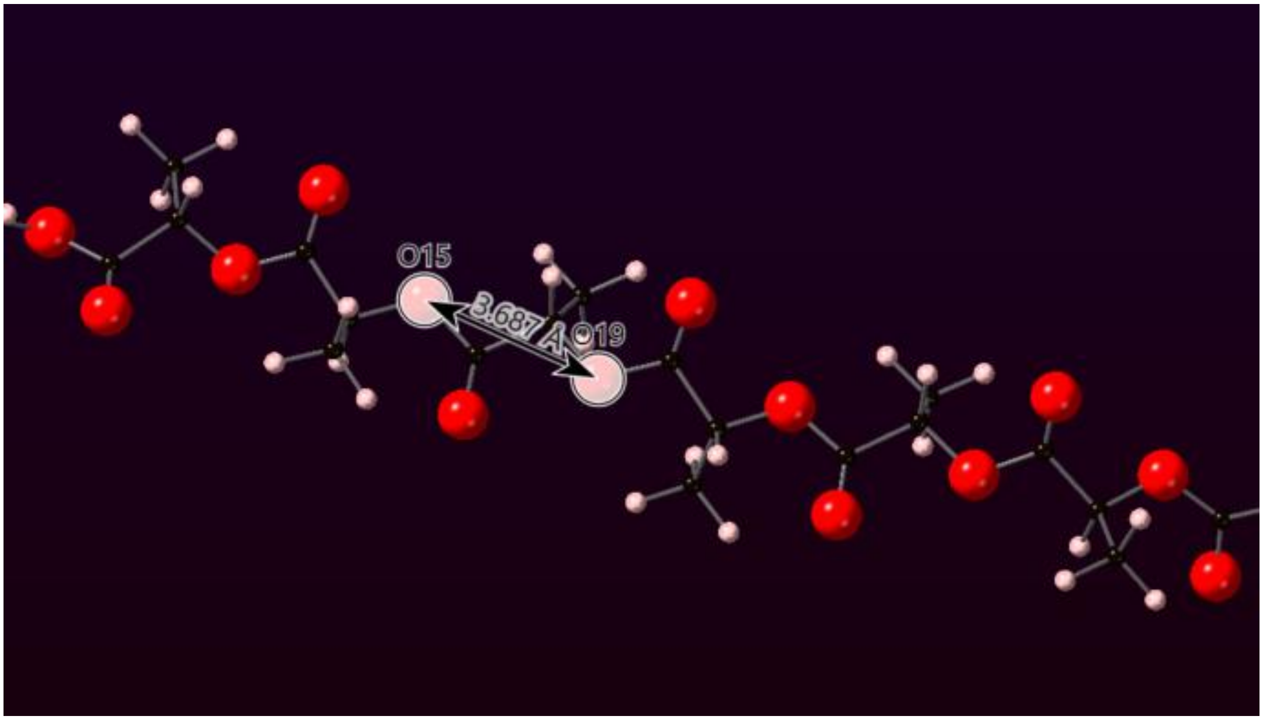

Supplementary Figure 25: Measurement of a PLLA polymeric unit from CrystalMaker® software. This measurement is used to calculate L_{max} as shown in the discussion (section a) in the main text.

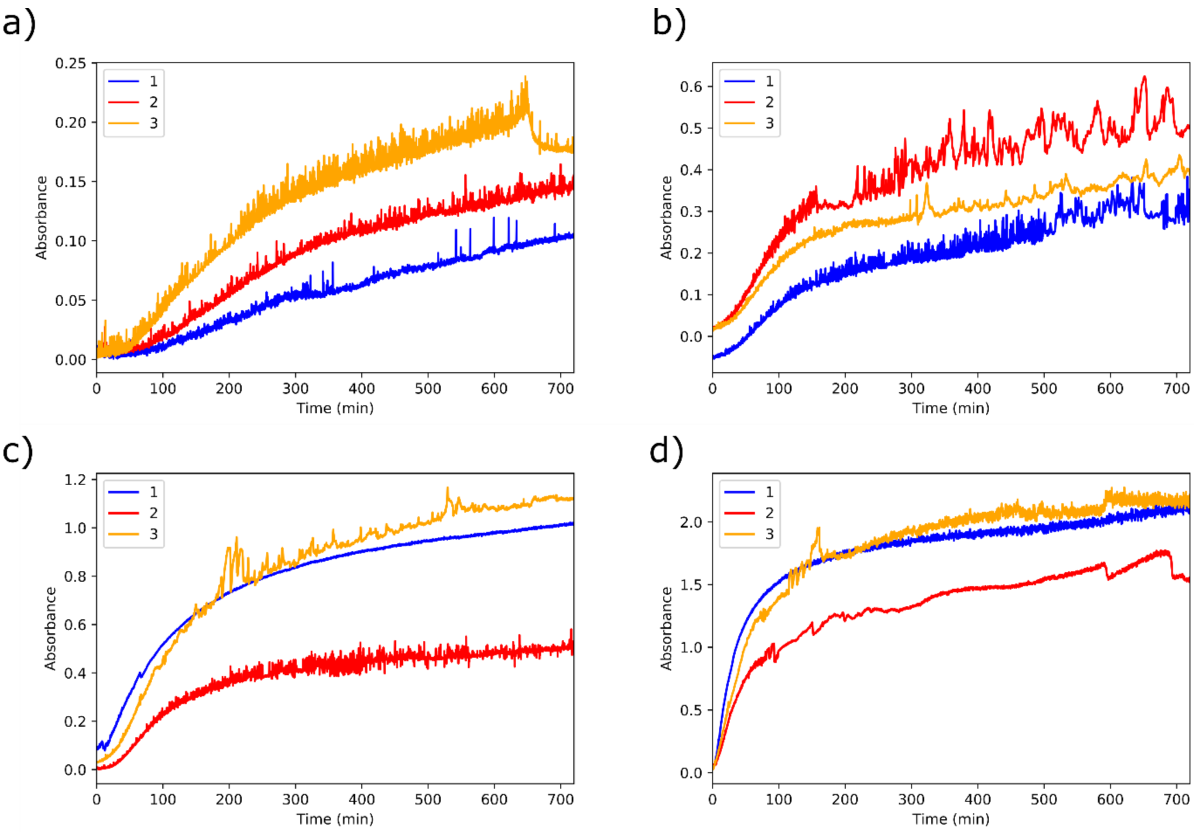

Supplementary Figure 26: Triplicate data from turbidity studies (600nm) for $PLLA_n$-*b*-PEG_{45} for (a) **9** (n=45), (b) **11** (n=60), (c) **12** (n=75), and (d) **15** (n=90) all at 10% solids w/w. Data presented in the main text is binned by a factor of 20 to reduce the noise. For the averaged data with error bars from the turbidity studies see Figure 1b in the main text. For the setup see the methods section.

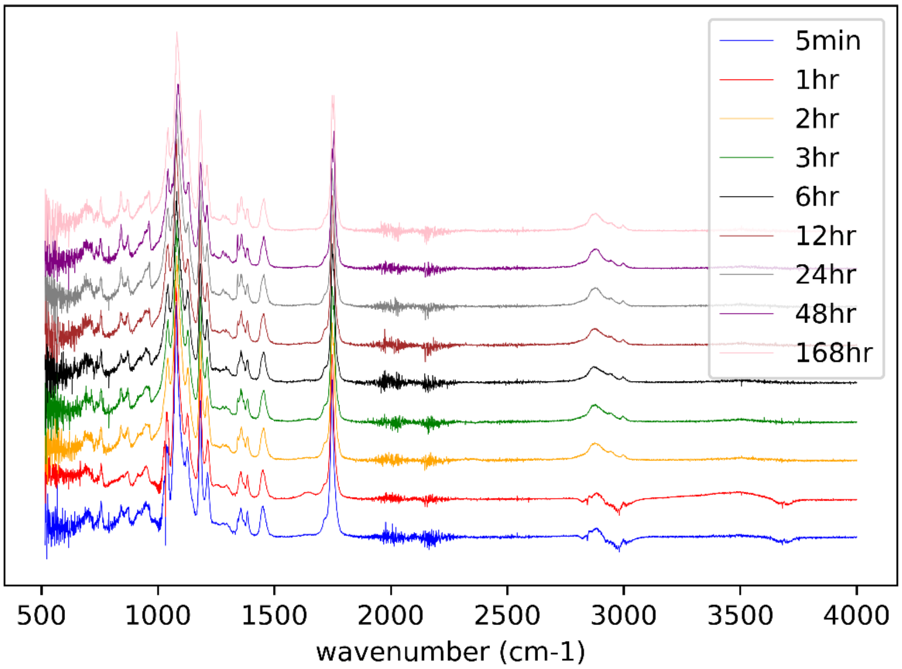

Supplementary Figure 27: Full FTIR spectra for time studies of $PLLA_{45}$-*b*-PEG_{45} (10% solids w/w **9**).

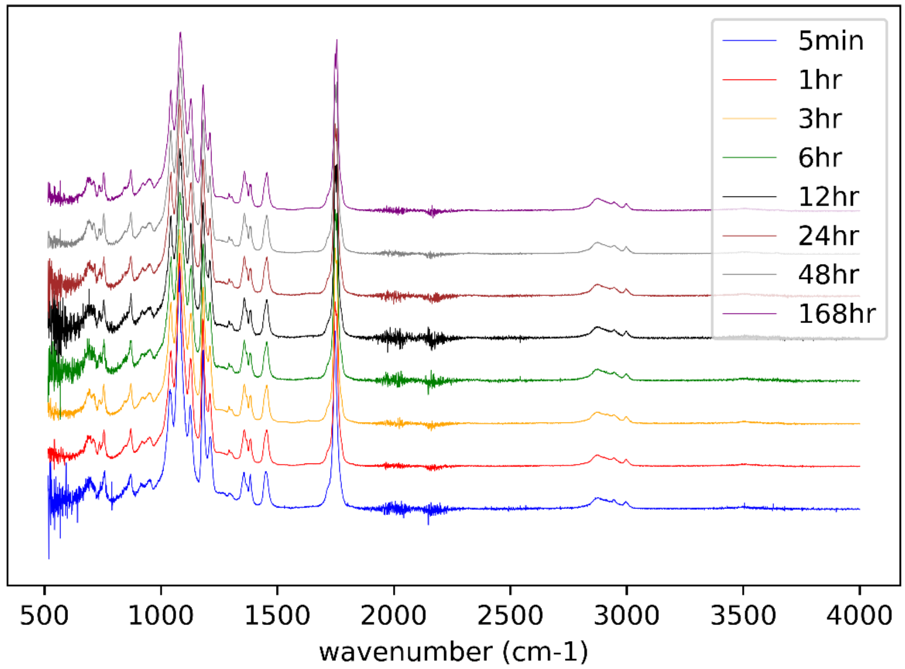

Supplementary Figure 28: Full FTIR spectra for time studies of $PLLA_{90}$-*b*-PEG_{45} (10% solids w/w **15**).

SUPPLEMENTARY REFERENCES

1. Yang, J.; Zhao, T.; Cui, J.; Liu, L.; Zhou, Y.; Li, G.; Zhou, E.; Chen, X., Nonisothermal crystallization behavior of the poly(ethylene glycol) block in poly(L-lactide)–poly(ethylene glycol) diblock copolymers: Effect of the poly(L-lactide) block length. *Journal of Polymer Science Part B: Polymer Physics* **2006,** *44* (22), 3215-3226.
2. Kricheldorf, H. R.; Meier-Haack, J., Polylactones, 22 ABA triblock copolymers of L-lactide and poly(ethylene glycol). *Die Makromolekulare Chemie* **1993,** *194* (2), 715-725.
3. Aldén, M.; Lydén, M.; Tegenfeldt, J., Effect of counterions on the interactions in solid dispersions between polyethylene glycol, griseofulvin and alkali dodecyl sulphates. *International Journal of Pharmaceutics* **1994,** *110* (3), 267-276.
4. Canning, S. L.; Smith, G. N.; Armes, S. P., A Critical Appraisal of RAFT-Mediated Polymerization-Induced Self-Assembly. *Macromolecules* **2016,** *49* (6), 1985-2001.
5. Mattia, E.; Otto, S., Supramolecular systems chemistry. *Nat. Nanotechnol.* **2015,** *10* (2), 111-119.
6. Marolf, D. M.; Jones, M. R., Measurement Challenges in Dynamic and Nonequilibrium Nanoscale Systems. *Analytical Chemistry* **2019**.
7. Nicolai, T.; Colombani, O.; Chassenieux, C., Dynamic polymeric micelles versus frozen nanoparticles formed by block copolymers. *Soft Matter* **2010,** *6* (14), 3111-3118.
8. Takahashi, R.; Miwa, S.; Sobotta, F. H.; Lee, J. H.; Fujii, S.; Ohta, N.; Brendel, J. C.; Sakurai, K., Unraveling the kinetics of the structural development during polymerization-induced self-assembly: decoupling the polymerization and the micelle structure. *Polym. Chem.* **2020,** *11* (8), 1514-1524.
9. Cheng, G.; Perez-Mercader, J., Polymerization-Induced Self-Assembly for Artificial Biology: Opportunities and Challenges. *Macromol. Rapid Commun.* **2019,** *40* (2), e1800513.
10. Varlas, S.; Foster, J. C.; O'Reilly, R. K., Ring-opening metathesis polymerization-induced self-assembly (ROMPISA). *Chem Commun (Camb)* **2019,** *55* (62), 9066-9071.
11. Penfold, N. J. W.; Yeow, J.; Boyer, C.; Armes, S. P., Emerging Trends in Polymerization-Induced Self-Assembly. *Acs Macro Letters* **2019,** *8* (8), 1029-1054.
